# Docosahexaenoic acid blocks progression of western diet-induced nonalcoholic steatohepatitis in obese *Ldlr^-/-^* mice

**DOI:** 10.1371/journal.pone.0173376

**Published:** 2017-04-19

**Authors:** Kelli A. Lytle, Carmen P. Wong, Donald B. Jump

**Affiliations:** 1 Nutrition Program, School of Biological and Population Health Sciences, Oregon State University, Corvallis, Oregon, United States of America; 2 Linus Pauling Institute, Oregon State University, Corvallis, Oregon, United States of America; Bambino Gesù Children's Hospital, ITALY

## Abstract

**Background:**

Nonalcoholic fatty liver disease (NAFLD) is a major public health concern in western societies. Nonalcoholic steatohepatitis (NASH), the progressive form of NAFLD, is characterized by hepatic steatosis, inflammation, oxidative stress and fibrosis. NASH is a risk factor for cirrhosis and hepatocellular carcinoma. NASH is predicted to be the leading cause of liver transplants by 2020. Despite this growing public health concern, there remain no Food and Drug Administration (FDA) approved NASH treatments. Using *Ldlr* ^*-/-*^ mice as a preclinical model of western diet (WD)-induced NASH, we previously established that dietary supplementation with docosahexaenoic acid (DHA, 22:6,ω3) attenuated WD-induced NASH in a prevention study. Herein, we evaluated the capacity of DHA supplementation of the WD and a low fat diet to fully reverse NASH in mice with pre-existing disease.

**Methods:**

*Ldlr* ^*-/-*^ mice fed the WD for 22 wks developed metabolic syndrome (MetS) and a severe NASH phenotype, including obesity, dyslipidemia, hyperglycemia, hepatic steatosis, inflammation, fibrosis and low hepatic polyunsaturated fatty acid (PUFA) content. These mice were randomized to 5 groups: a baseline group (WDB, sacrificed at 22 wks) and 4 treatments: 1) WD + olive oil (WDO); 2) WD + DHA (WDD); 3) returned to chow + olive oil (WDChO); or 4) returned to chow + DHA (WDChD). The four treatment groups were maintained on their respective diets for 8 wks. An additional group was maintained on standard laboratory chow (Reference Diet, RD) for the 30-wk duration of the study.

**Results:**

When compared to the WDB group, the WDO group displayed increased hepatic expression of genes linked to inflammation (*Opn*, *Il1rn*, *Gdf15*), hepatic fibrosis (collagen staining, *Col1A1*, *Thbs2*, *Lox*) reflecting disease progression. Mice in the WDD group, in contrast, had increased hepatic C_20-22_ ω3 PUFA and no evidence of NASH progression. MetS and NASH markers in the WDChO or WDChD groups were significantly attenuated and marginally different from the RD group, reflecting disease remission.

**Conclusion:**

While these studies establish that DHA supplementation of the WD blocks WD-induced NASH progression, DHA alone does not promote full remission of diet-induced MetS or NASH.

## Introduction

The Centers for Disease Control and Prevention estimate that nearly 80 million adults [1] and 13 million children [2] in the US are obese. Nonalcoholic fatty liver disease (**NAFLD**) is strongly associated with obesity [3, 4]; and is the most common chronic fatty liver disease in developed countries [5]. NAFLD is defined as excessive neutral lipid (triglycerides and cholesterol esters) deposition in the liver, i.e., hepatosteatosis [6, 7]. The top 4 risk factors for NAFLD are obesity, dyslipidemia, type 2 diabetes mellitus (**T2DM**) and metabolic syndrome (**MetS**) [8, 9].

NAFLD is a continuum of diseases ranging from benign fatty liver to primary hepatocellular cancer (**HCC**). Approximately 30% of the US population is estimated to have some form of chronic fatty liver disease [10]. Ten to 30% of NAFLD patients develop nonalcoholic steatohepatitis (**NASH**) [10, 11], the progressive form of the disease. NASH is characterized by hepatic steatosis, inflammation, oxidative stress and injury. Excessive damage to the liver resulting from NASH promotes tissue repair involving deposition of extracellular matrix components (**ECM**), consisting of collagens, elastin and other proteins, i.e., fibrosis. NASH has high prevalence (≥60%) in the T2DM population [12]; and is recognized as a risk factor for cardiovascular disease [13–15]. NASH patients also have higher mortality rates than NAFLD patients; and both have a higher mortality rates than the general population. Twenty to 30% of NASH patients progress to cirrhosis. Over a 10 year period, cirrhosis and liver related deaths occur in 20% and 12% of NASH patients, respectively [16]. By the year 2020, cirrhosis resulting from NASH is projected to be the leading cause of liver transplantation in the United States [17]. Given the increasing prevalence of NASH and its adverse clinical outcomes, NASH is considered a major public health concern [18].

Diet, genetics and lifestyle contribute to the onset and progression of NAFLD and NASH. While the best strategy for managing NASH has yet to be defined [18–21], current strategies focus on lifestyle management, including exercise and diet. Diets recommended for NAFLD therapy are low in fat, cholesterol & simple sugar [19, 22–34]. We have examined the effect of ω3 PUFA in NASH therapy because ω3 PUFA lower blood triglycerides, reduce hepatic fatty acid synthesis and inflammation and increase triglyceride catabolism [35–41]. Moreover, NAFLD patients consuming a western-style diet have low hepatic PUFA content, and the loss of hepatic PUFA worsens as patients transition from benign hepatosteatosis to advanced NASH [42–44]

Our focus is on attenuating the impact of the western diet (**WD**) on NASH; the WD is a leading culprit in the obesity epidemic [40–42, 45–50]. The WD is moderately high in fat (saturated and trans-fat), simple sugar (sucrose & fructose) and cholesterol and low in essential PUFA, i.e., linoleic acid (18:2,ω6) and γ-linolenic acid (18:3,ω3). Despite the promise of ω3 PUFA in NAFLD therapy, clinical studies using ω3 PUFA supplements have yielded mixed results [29, 51–56]. Studies using either fish oil or Lovaza [~50:50 mix of eicosapentaenoic acid (20:5,ω3, **EPA**) & docosahexaenoic acid (**DHA**, 22:6,ω3) ethyl esters (at ~4 g/d, GSK] have reported success in reducing steatosis, but little success in improving fibrosis scores [55–58]. EPA-ethyl esters (1.8 and 2.7 g/d) fail to reduce steatosis or fibrosis [57], a finding that recapitulates our observations using the WD-*Ldlr*^*-/-*^ mouse model for NASH [40]. The failure of EPA to lower liver fat and fibrosis in humans is likely due to the poor conversion of EPA to DHA in humans [58]. Both EPA and DHA suppress the expression of elongases & desaturases required for PUFA synthesis [37]. DHA is a major bioactive ω3 PUFA accumulating in tissues and is likely responsible for many of the beneficial effects of ω3 fatty acids seen *in vivo*.

Our previous studies using ω3 PUFA focused on NASH prevention [40, 41, 59]. We established that DHA interferes with transforming growth factor (**TGFβ**) signaling and attenuates WD-induced hepatic fibrosis in a prevention study [60]. TGFβ is a major regulator of hepatic fibrosis [61]. More recently, we focused on treating obese mice with pre-existing NASH [50], a likely scenario seen clinically. Herein, we evaluated the capacity of DHA to promote NASH remission in mice with pre-existing NASH. Previous animal studies have shown that hepatic fibrosis induced by intraperitoneal CCL4 injection, bile duct ligation or a choline-methionine deficient diet was reversed within 4, 12 and 2 wks, respectively [62–64] after removal of the pro-fibrotic agent. These models, however, lack the severe obese and diabetic phenotype associated with human NASH. We predict that the obese-T2DM phenotype will negatively affect NASH remission.

Our preclinical mouse model uses the WD to induce a severe NASH and MetS-like phenotype in *Ldlr*^*-/-*^ mice. These mice are obese, hyperglycemic, dyslipidemic and endotoxinemic. Their livers are fatty (steatotic), inflamed and fibrotic [65–67]. Like humans with NASH [42], WD-fed mice have a significant reduction in hepatic PUFA with ω3 PUFA being more affected than ω6 PUFA. Herein, we tested the hypothesis that dietary DHA will overcome the impact of WD and fully reverse NASH, including fibrosis, in mice with pre-existing disease. The outcome of our studies reveal the strengths and limitations of using dietary ω3 PUFA in NASH therapy.

## Materials and methods

### Animals and diets

This study was carried out in strict accordance with the recommendations in the Guide for the Care and Use of Laboratory Animals of the National Institutes of Health. All procedures for the use and care of animals for laboratory research were approved by the Institutional Animal Care and Use Committee at Oregon State University (Permit Number: A3229-01). Male *Ldlr*^*-/-*^ mice [B6;129S7-*Ldlr*^*1Her*^/J, stock# 002207, purchased from Jackson Labs] were individually housed, maintained on a 12-hour light/dark cycle; and mice were acclimatized to the animal facilities at OSU for 1-week before proceeding with experiments. At the termination of the studies, all mice were fasted overnight (1800 h to 0800 h) prior to euthanasia by CO_2_ administration and exsanguination; blood and liver were collected as previously described [66].

**Fig 1:**
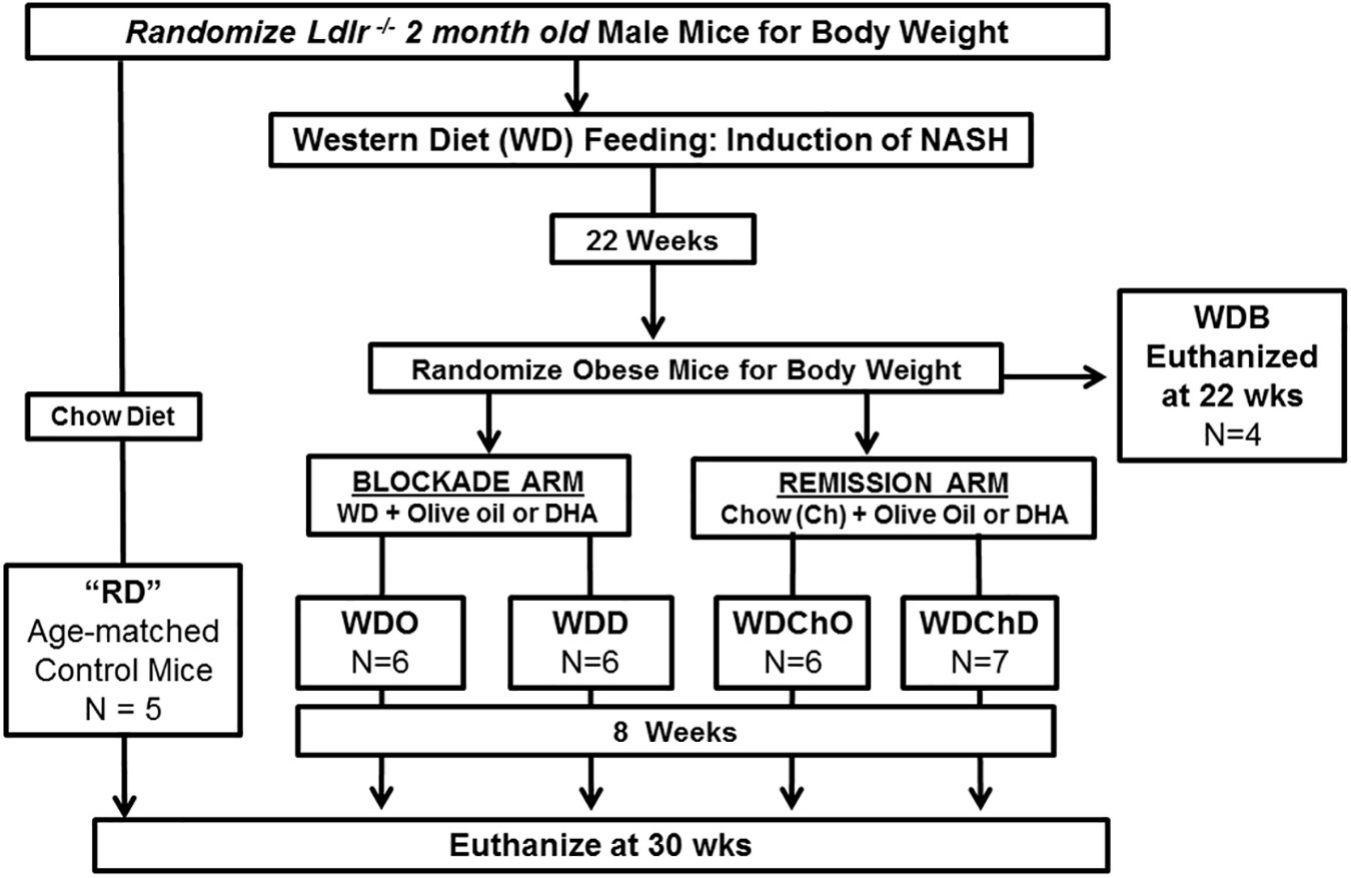
Study design to examine the reversibility of NASH in male *Ldlr* ^*-/-*^ mice. The study design included two treatment arms; a blockade arm and a remission arm. Mice were initially randomized to 2 groups: **a**) mice maintained on chow for 30 weeks, reference diet (**RD,** n = 5); **b**) mice maintained on the western diet (**WD**) for 22 wks. After 22 wks on the WD, four mice were euthanized for blood and liver collection; these mice served as the baseline group (**WDB,** n = 4). The remaining WD-fed mice were randomized to 4 groups: **1**) mice fed the western diet + olive oil for 8 wks (**WDO,** n = 6), **2**) mice fed the WD + DHA for 8 wks (**WDD,** n = 6); **3**) mice fed the chow diet + olive oil for 8 wks (**WDChO,** n = 6); **4**) mice fed the chow diet + DHA for 8 wks (**WDChD,** n = 7). At termination of the study, mice in the RD, WDO, WDD, WDChO and WDChD groups were euthanized for liver and blood collection.

At 10 weeks of age male mice were randomized to 2 treatment groups; 8 mice were maintained on Purina Pico Lab Diet 5053 *ad libitum* for 30 wks [(Reference Diet, (**RD)**], while 40 mice were fed the Western Diet (**WD**, Research diets D12079B) *ad libitum* for 22 wks (**Fig 1**). At 22 wks on the WD, obese mice (average weight 38.3 ± 2.3 g) were randomized to 5 groups: Group 1 mice were euthanized and served as the WD-baseline group [**WDB**]; Group 2 mice were fed *ad libitum* the WD supplemented with olive oil for 8 wks [**WDO**]; Group 3 mice were fed the WD supplemented with DHA for 8 wks [**WDD];** Group 4 mice were fed the Purina Pico Lab Diet 5053 chow diet supplemented with olive oil for 8 wks (**WDChO**); Group 5 mice were fed the Purina Pico Lab Diet 5053-chow diet supplemented with DHA for 8 wks (**WDChD**).

DHA (DHASCO, DSM, 40% DHA as triglyceride) was added to the WD (**WDD**) and chow (**WDChD**) diets so that DHA was at 2% total energy. This level of DHA is comparable to that used in ω3-PUFA therapy where humans are prescribed Lovaza (GlaxoSmithKline: EPA- & DHA-ethyl esters; at 4 g/day) to treat hypertriglyceridemia [68, 69]. Olive oil was added to the WD (**WDO**) and chow (**WDChO**) diets to ensure all diets were isocaloric. Previous studies established that addition of olive oil to the WD had no impact on the health status of WD-fed mice [38, 40, 59].

A power calculation (http://www.dssresearch.com/toolkit/spcalc/power_a1.asp) was carried out with the following parameters: difference between the test (test value = 8) and control (control value = 4) i.e., mean difference is 2-fold; standard deviation 20% of the mean; 95% confidence, the statistical power for 4 and 6 animals (sample size) was 99.1% and 99.9%, respectively. We initially started with 8 mice/group, but some mice were euthanized prematurely due to severe dermatitis. As such, the RD, WDB, WDO, WDD, WDChO and WDChD had 5, 4, 6, 6, 6, 7 mice, respectively.

### RNA extraction and qRT-PCR

RNA was extracted from livers using Trizol (Life Technologies) as described [70], and quantified spectrophotometry using a nanodrop-1000. qRT-PCR was performed using the 7900HT fast machine from Applied Bio-systems as previously described [70].

qRT-PCR arrays were used to profile the expression of genes involved in fibrosis (Mouse Fibrosis RT^2^ Profiler PCR Array) and genes related to cytokines (Mouse Common Cytokines RT^2^ Profiler PCR Array) according to the manufacturer’s protocol (Qiagen, Valencia, CA, USA). Gene expression was normalized to Hsp90ab1, and relative quantification was determined using the ΔΔCt method.

### Hepatic lipid composition

Hepatic lipids were extracted as previously described [71]. Total lipid extracts were saponified and the fatty acids were methylated. Fatty acid methyl esters were separated and quantified by gas chromatography (**GC**) [71]. GC standards were purchased from Nu-Chek Prep Inc. Hepatic protein content was measured using Quick Start Bradford Reagent (Bio-Rad) and bovine serum albumin (Sigma-Aldrich) as a standard.

### Measurement of plasma and hepatic osteopontin

Plasma osteopontin (**OPN**) was quantified by Elisa (R&D systems) according to the manufactures protocols. Hepatic OPN was quantified by immunoblot analysis of whole cell extracts using mouse antibodies against Opn (R & D Systems) and vinculin (Millipore) as described previously [40, 60].

### Plasma and hepatic measures

Plasma triglycerides, total and free cholesterol and glucose were measured using kits obtained from Wako. Plasma aspartate amino transferase **(AST)** and alanine amino transferase **(ALT)** were measured using kits from Thermo Fischer Scientific. Plasma Toll Like receptor **(TLR)**2 and TLR4 agonist activity was measured using Hek-Blue cell systems from Invivogen.

### Liver histology

Liver (~100 mg) was fixed in buffered-formalin, paraffin embedded, sliced, and stained with hematoxylin-eosin or trichrome (Nationwide Histology, Veradale, WA). Each slide contained 2–4 slices/liver. Steatosis and fibrosis was seen consistently on all liver sections from the same animal. Photomicrographs of liver sections shown in the figures are representative of all livers within each group.

### Heat maps, volcano plots and statistical analysis

Heat maps were prepared using data on body weight, plasma (glucose, lipids, ALT, AST, TLR2 and TLR4 agonist, cholesterol, triglycerides), and hepatic (triglycerides, cholesterol, fatty acid profiles and gene expression) parameters. The data was analyzed using the statistical package in MetaboAnalyst 3.0 [http://www.metaboanalyst.ca/MetaboAnalyst/] [72]. The analyses generated heat maps, volcano plots, correlation analyses and ANOVA with Tukey’s HSD Post-hoc test. We also used a separate online statistical package [http://vassarstats.net/] for one-way ANOVA with Tukey’s HSD Post-hoc test of specific features to detect significant differences between groups when more than two groups were included in the analysis. Student’s t-test was used when only two groups were being compared and non-parametric tests were used when unequal variance as determined by f-test was detected between two groups. A p-value ≤0.05 was considered statistically different. All values are reported as mean ± SD.

## Results

### DHA halts NASH progression in *Ldlr* ^*-/-*^ mice with pre-existing disease

**Fig 2:**
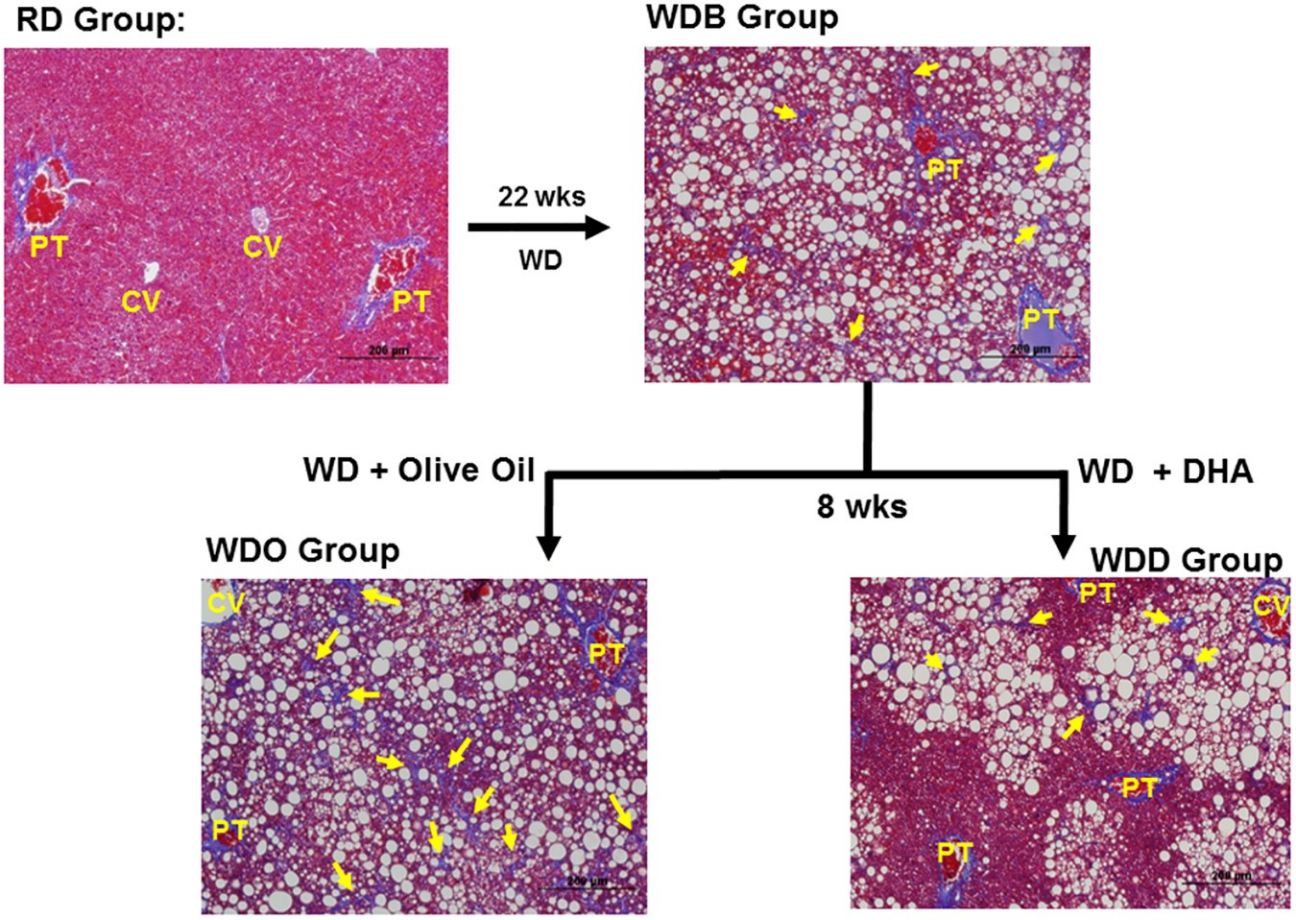
Diet effects on hepatic morphology: Blockade arm. Livers sections from the 4 treatment groups (RD, WDB, WDO and WDD) in the Blockade Arm were stained with trichrome and photographed at 4x. Lipid droplets appear as white circles, while branching fibrosis appears as blue strands (yellow arrows) in the trichrome stained liver sections. The slides are representative of multiple sections of each liver and all livers in each group. PT, portal track; CV, central vein.

*Ldlr* ^*-/-*^ mice fed the WD for 22 wks (**WDB group**) are obese, hyperglycemic, dyslipidemic and display evidence of significant hepatic injury (blood ALT and AST) and systemic inflammation (plasma TLR2 and TLR4 agonist) (**Table 1**). Livers of these mice were increased in size relative to body weight and contain elevated levels of cholesterol and saponifiable fatty acids. Saponifiable fatty acids are derived from neutral lipids (triacylglycerides and diacylglycerides & cholesterol esters) and phosphoglycerolipids; and represent a more complete measure of changes in hepatic fats associated with hepatosteatosis, than measuring triglycerides alone. Hepatic histology of mice in the WDB group revealed severe steatosis (lipid droplets, which appear as white vacuoles) as well as mild branching fibrosis, which appears as blue branching strands in trichrome stained livers (**Fig 2**).

**Table 1:** Effect of DHA on NASH remission: Anthropometric, plasma and hepatic features^1^.

	RD	WDB	WDD	WDO
**Features**				
Body Weight, g	25.0 ± 3.0^a^	38.3 ± 2.3^b^	41.0 ± 5.1^b^	41.9 ± 3.1^b^
**Plasma**				
Glucose, mg/dL	126 ± 66^a^	356 ± 102^b^	330 ± 102^b^	408 ± 146^b^
Triglycerides, mg/dL	47 ± 28^a^	277 ± 36^b^	228 ± 98^b^	517 ± 218^c^
Cholesterol, mg/dL	143 ± 54^a^	760 ± 118^b^	878 ± 198^b^	1398 ± 393^c^
ALT, U/L	9.7 ± 2.0^a^	52.0 ± 16.6^b^	43.0 ± 15.2^b^	67.1 ± 15.5^b^
AST, U/L	22.1 ± 5.0^a^	57.7 ± 27.3^b,c^	41.4 ± 10.5^b^	85.2 ± 17.2^c^
TLR2 Agonist, U/mL	5.1 ± 2.9^a^	9.4 ± 2.9^a^	3.7 ± 3.1^a^	14.5 ± 9.7^b^
TLR4 Agonist, U/mL	17.8 ± 7.1^a^	36.5 ± 6.3^b^	32.3 ± 13.3^b^	37.5 ± 12.7^b^
**Liver**				
Liver weight, g	1.15 ± 0.14^a^	2.01 ± 0.3^b^	1.85 ± 0.5^b^	2.6 ± 0.7^b^
Liver Weight/BW, g%	4.6 ± 0.2^a^	5.3 ± 0.4^b^	4.4 ± 0.7^a^	6.2 ± 1.2^c^
Cholesterol, μg/g protein	27.4 ± 14.5^a^	31.7 ± 9.1^b^	36.1 ± 14.7^a^	47.8 ± 14.3^b^
Fatty Acyls, μmol/g protein	2.4 ± 0.6^a^	5.0 ± 1.2^b^	3.4 ± 0.9^a^	5.3 ± 0.7^b^

^1^Results are presented as mean ± SD, RD, n = 5; WDB, n = 4; WDD, n = 6; WDO, n = 6. Statistical analysis used ANOVA plus Tukey’s HSD to establish statistical significance. Labeled means in a row with superscripts without a common letter differ, P ≤ 0.05.

Maintaining the mice on the WD for an additional 8 wks (**WDO group**) does not significantly increase body weight or blood glucose, but significantly increased plasma lipids (triglycerides and cholesterol) and modestly increased hepatic injury and systemic inflammation (**Table 1**). Liver weight, cholesterol and fatty acyls are higher in the WDO group versus the WDB group. Extensive branching fibrosis is apparent throughout the liver in the WDO group, reflecting disease progression, particularly in fibrosis.

Mice switched from the WD to the WD supplemented with DHA (**WDD group**) resulted in no change body weight, blood glucose or TLR4 agonist, but a significant reduction in plasma TLR2 agonists, when compared to the WDB group. When compared to the WDO group, however, mice fed the WDD had significantly lower plasma triglycerides, cholesterol and AST, but not ALT levels. Hepatic weight, cholesterol and fatty acyls were all significantly lower in the WDD group, compared to the WDB and WDO groups. Histological examination revealed hepatic lipid droplets were absent from the periportal region, but clustered around the central vein. This pattern of steatosis may reflect increased triglyceride catabolism and fatty acid oxidation in the periportal region. While branching fibrosis was absent in livers in the WDD group, small isolated patches of fibrosis were located near large lipid droplets. The absence of extensive branching fibrosis in the WDD group, as seen in the WDO group, likely reflects inhibition of ECM formation during the 8 wk WDD feeding.

### Impact of diet on hepatic fatty acid composition in the blockade arm

**Fig 3:**
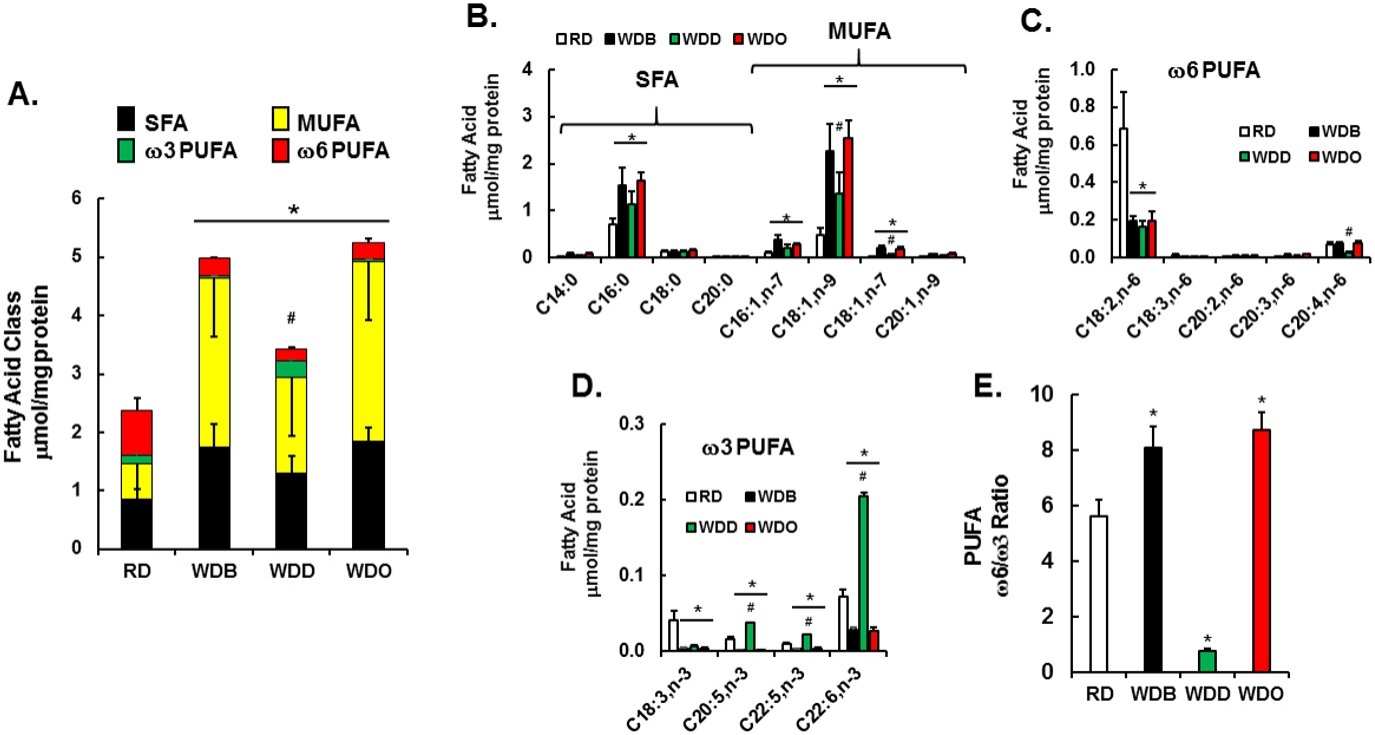
Analysis of hepatic fatty acids: Blockade arm. Hepatic fatty acids were quantified as described in Materials and Methods. **A:** The sum of fatty acids in the 4 lipid classes (saturated (SFA), monounsaturated (MUFA), ω3 and ω6 polyunsaturated fatty acids (PUFA) is presented in a stacked histogram. **B-D**: Specific fatty acids in each class are quantified and presented as μmol/mg protein. **E:** The mole ratio of the sum of ω3 and ω6 PUFA. Mean ± SD with 4–7. *, p<0.05 versus the RD group; #, p<0.05 versus the WDO group using one-way ANOVA.

The WD induces a significant increase in total fatty acyls (**Table 1, Fig 3**), composed mainly of saturated (**SFA**) and monounsaturated (**MUFA**) fat (**Fig 3A & 3B**). Adding DHA to the WD had no significant effect on hepatic SFA, but significantly lowered MUFA, including 16:1,ω7; 18:1,ω7 & 18:1,ω9. These fatty acids are derived from the diet and also synthesized endogenously by stearoyl CoA desaturase (Scd1) mediated desaturation of 16:0 and 18:0. DHA suppresses hepatic Scd1 expression in WD-fed mice [40].

Although the WD is an essential fatty acid sufficient diet, the essential fatty acids (18:2,ω6 & 18:3,ω3) represent a low percentage of total dietary fat in the WD [40]. Hepatic levels of ω3 and ω6 PUFA were significantly reduced in mice fed the WD-fed (**Fig 3C & 3D**). While hepatic 18:2,ω6 was reduced by ~60% in the WDB and WDO groups, its downstream product, i.e., arachidonic acid (20:4,ω6), was not reduced, when compared to the RD group. In contrast, the ~60% reduction in hepatic 20:4,ω6 in WDD-fed mice is likely due to suppressed PUFA synthesis, since DHA suppresses expression of Fads1, Fads2 and Elovl5 [40]. Increased hepatic EPA, DPA and DHA in the WDD-fed group is consistent with previous studies showing that dietary DHA increased hepatic 20:5,ω3 and 22:5,ω3, likely through retroconversion. While the WD lowers hepatic ω3 and ω6 PUFA content and also significantly increased hepatic ω6/ω3 PUFA ratio by ~35%, dietary DHA significantly decreased the ω6/ω3 PUFA ratio by ~80% when compared to the RD group (**Fig 3E**).

### Overview of the capacity of DHA to block WD-induced NASH progression

**Fig 4:**
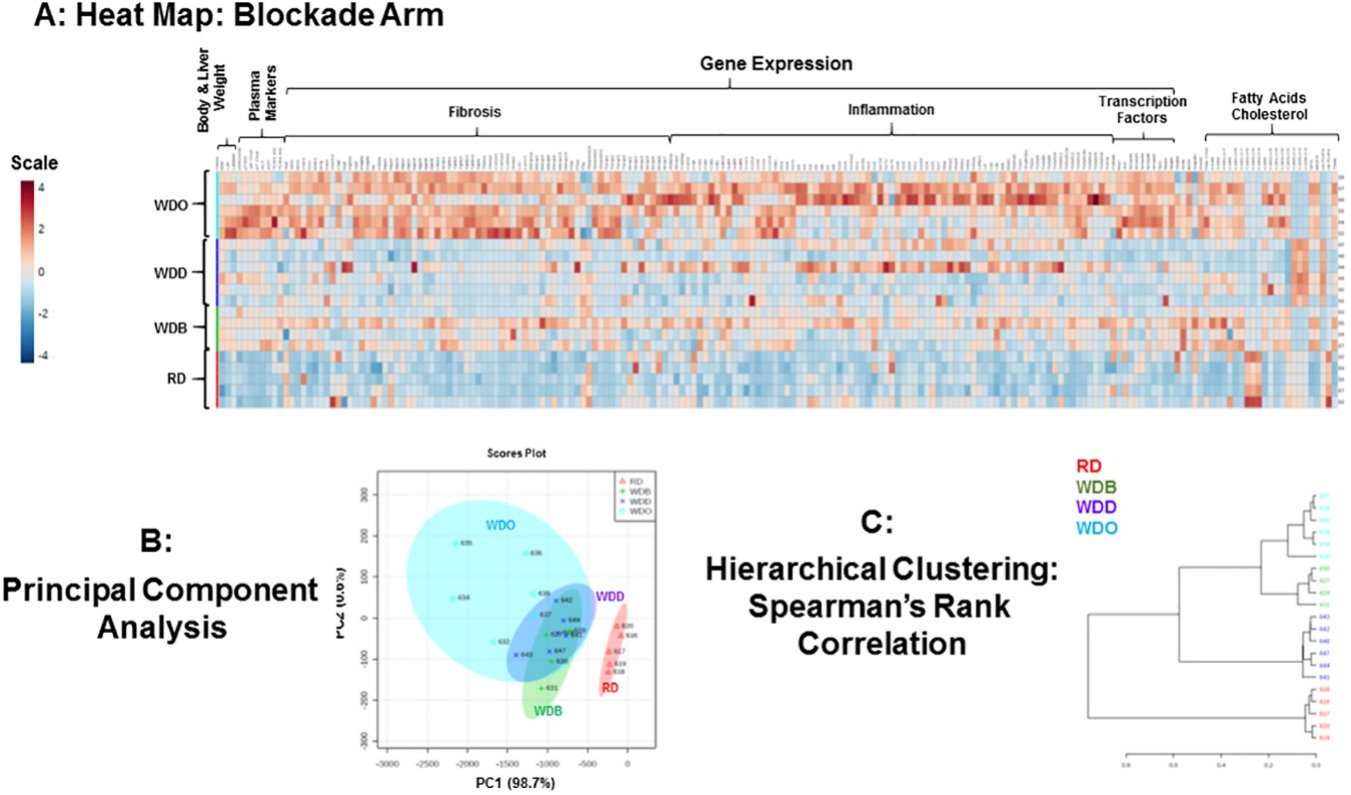
Overview of diet effects on anthropometric, plasma, hepatic gene expression and lipid parameters in the blockade arm. All anthropometric, plasma, gene expression and hepatic lipid features were assembled into an excel spreadsheet for each mouse in each of the 4 groups (RD, WDB, WDO, WDD). The data was analyzed using (http://www.metaboanalyst.ca/MetaboAnalyst/) to create a heat map **[A]** and carry out a principal component analysis **[B]** and hierarchical clustering using Spearman’s ranked correlation **[C].** The heat map is a visualization of the changes in abundance/level of features for each animal. Animal identification numbers are listed on the right side of the heat map. The color ranges from deep orange (high abundance or level) to deep blue (low abundance or level); white represents no change.

We next examined diet effects on two major pathways contributing to NASH progression, i.e., inflammation and fibrosis. Accordingly, we used commercially available qRTPCR arrays for an in-depth analysis of inflammation (cytokines) and fibrosis. Results from the gene expression analysis, along with the anthropometric, plasma and fatty acid data (**Table 1 and Fig 3**) were assembled in a spreadsheet and analyzed using the statistical package in MetaboAnalyst 3.0. This analysis allowed us to generate a heat map, a principal component (**PCA**) and a hierarchical cluster analysis (**HCA**) (**Fig 4**) [72].

The analysis revealed broad effects of the WD on whole body, plasma and hepatic features. Moreover, most features increased from 22 wks (WDB) to 30 wks (WDO) on the WD, reflecting disease progression. Features that decreased included the essential ω3 and ω6 PUFA and their C_18-22_ PUFA products (**Fig 3**). The PCA analysis shows that the RD group differs from all WD-fed groups; and that the number and level of changed features increased from the WDB to the WDO group, again reflecting disease progression.

Particularly relevant is the finding that the WDB and WDD groups overlap reflecting little difference in features when compared to RD group (**Fig 4B**). The key difference between these two groups is that mice in the WDB groups were euthanized at 22 wks, while mice in the WDD group were placed on the WD containing DHA (WDD) diet at 22 wks and euthanized 8 wks later (**Fig 1**). The hierarchical clustering analysis established that the WDB, WDO and WDD groups cluster separately from the RD group, while the WDD group segregates as a separate subgroup.

### Impact of DHA on hepatic gene expression in the blockade arm

**Fig 5:**
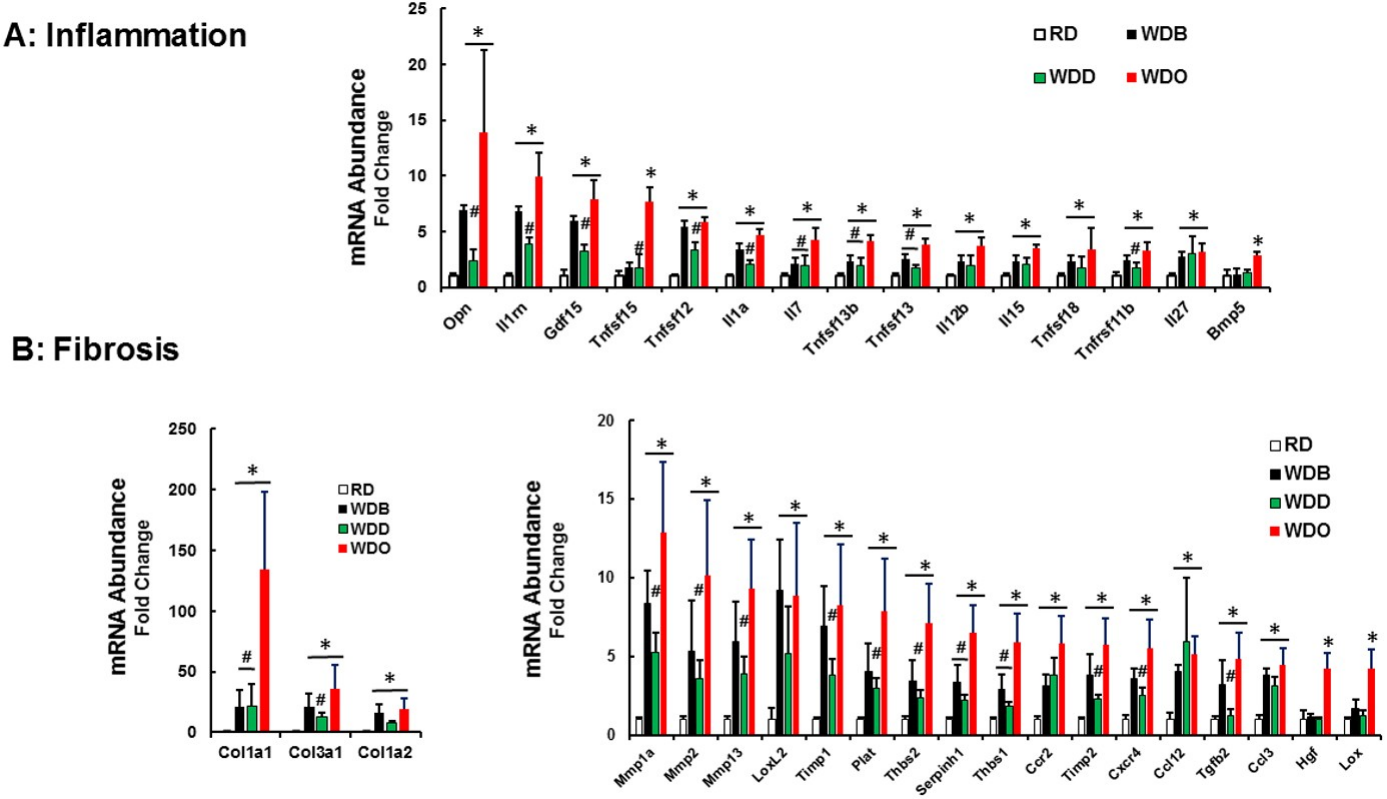
**Diet effects on hepatic expression of proteins linked to inflammation [A] and fibrosis [B] in the blockade arm.** Hepatic mRNA abundance was quantified as described in Materials and Methods. Results are represented as mRNA Fold Change; N = 4–7; mean ± SD; *, p<0.05 versus the RD group; #, p<0.05 versus the WDO group; one-way ANOVA.

Of the 84 transcripts in the cytokine array, 15 transcripts were induced ≥ 3-fold by the WD (**Fig 5A**). Many of these transcripts increased from 22 to 30 wks of WD feeding reflecting disease progression (e.g., *Opn*, *IL1rn*, *IL7*), while other transcripts were significantly induced only at 30 wks, reflecting late onset in the course of the disease (*Tnfsf15*, *Bmb5*). For all genes examined, DHA (WDD) either knocked down expression (*Opn*, *IL1rn*, *Gdf15*, *Tnfsf12*) or blocked further increases in transcript abundance (*IL7*, *IL15*, *Bmp5*).

**Fig 6:**
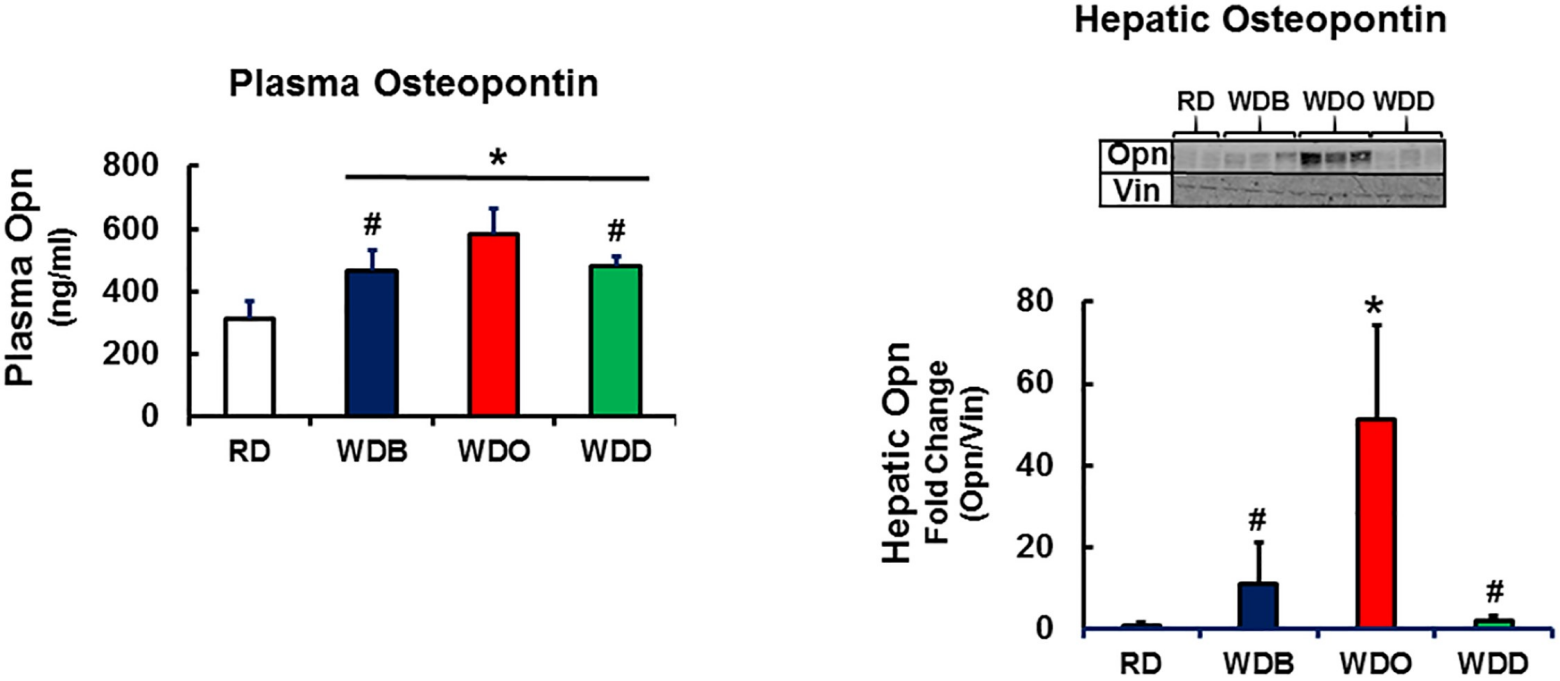
Quantitation of plasma and hepatic osteopontin (Opn). Mouse plasma osteopontin levels were quantified by ELISA (R & D Systems). Results are expressed as Opn, ng/ml of plasma, N = 4–7; mean + SD; *, p<0.05 versus the RD group; #, p<0.05 versus the WDO group; one-way ANOVA. Hepatic Opn was quantified by immunoblot analysis of whole cell hepatic extracts as described previously. The mouse antibodies use in the analysis were anti-Opn (R & D Systems) and vinculin (Millipore); vinculin (Vin) was a loading control. Results were quantified by Licor Odyssey [40, 60] and expressed as abundance of Opn/Vin. Extracts from 3 separate livers in each group were examined by immunoblot analysis. Results are expressed as mean + SD; *, p<0.05 versus the RD group; #, p<0.05 versus the WDO group; one-way ANOVA.

Of the cytokine transcripts examined, Opn stands out as being highly expressed and well-regulated by diet in the liver (**S1 Fig**). *Opn* is a phosphoprotein cytokine secreted from many cell types; and its plasma level has been linked to hepatic fibrosis [73]. Accordingly, we quantified plasma and hepatic Opn levels (**Fig 6**). Plasma Opn was induced ~40% after 30 wks on the WD. Plasma Opn levels in the WDB and WDD groups are comparable and both are lower than that seen in the WDO group. Hepatic Opn protein, in contrast, is low in the RD and WDD groups, but well induced (10- and 50-fold) in the WDB and WDO groups, respectively. Hepatic Opn protein parallels changes in hepatic Opn mRNA.

Additional gene expression markers of inflammation (*Mcp1*, *Tlr4*, *Tnfα*, *CD68*) and oxidative stress (*Nox2*) were examined by qRTPCR *(***S2 Fig***)* [50]. We previously reported that the WDO diet induced and WDD diet suppressed plasma levels of TNF*α* and agonist for TLR2 and TLR4 [60]. Most of these markers increased from 22 to 30 wks on the WDO diet providing further evidence of disease progression. TNF*α*, in contrast, was well induced by 22 wks (~6-fold) and showed no further increase at 30 wks on the WD. A complete list of features that increased (≥ 2-fold) or decreased (≥ 50%) significantly (*p-value* ≤ 0.05) in the comparison of the RD and WDO groups is in **S1 Table**.

Of the 84 transcripts on the fibrosis qRTPCR array, 20 were induced >3-fold (**Fig 5B)**. The most striking evidence of progression is the induction of Col1A1, the major collagen subtype induced in rodent and human NASH [60, 74, 75]. Of the 3 collagen subtypes examined, Col1A1 was expressed at the lowest level in the RD group (**S1 Fig**) and shows the highest fold change of any collagen subtype examined. While many of the transcripts linked to fibrosis were well-induced by 22 wks, the diet effects on hepatic growth factor (***Hgf***) and lysyl oxidase (***Lox***) were only apparent after 30 wks on the WDO diet. In nearly all cases, DHA knocked down or prevented further induction of the transcripts linked to fibrosis, reflecting a robust anti-fibrotic effect of DHA. These changes in expression of fibrosis linked genes correlated with the reduction in ECM staining in the WDD group (**Fig 2)**.

### DHA blocks disease progression

**Fig 7:**
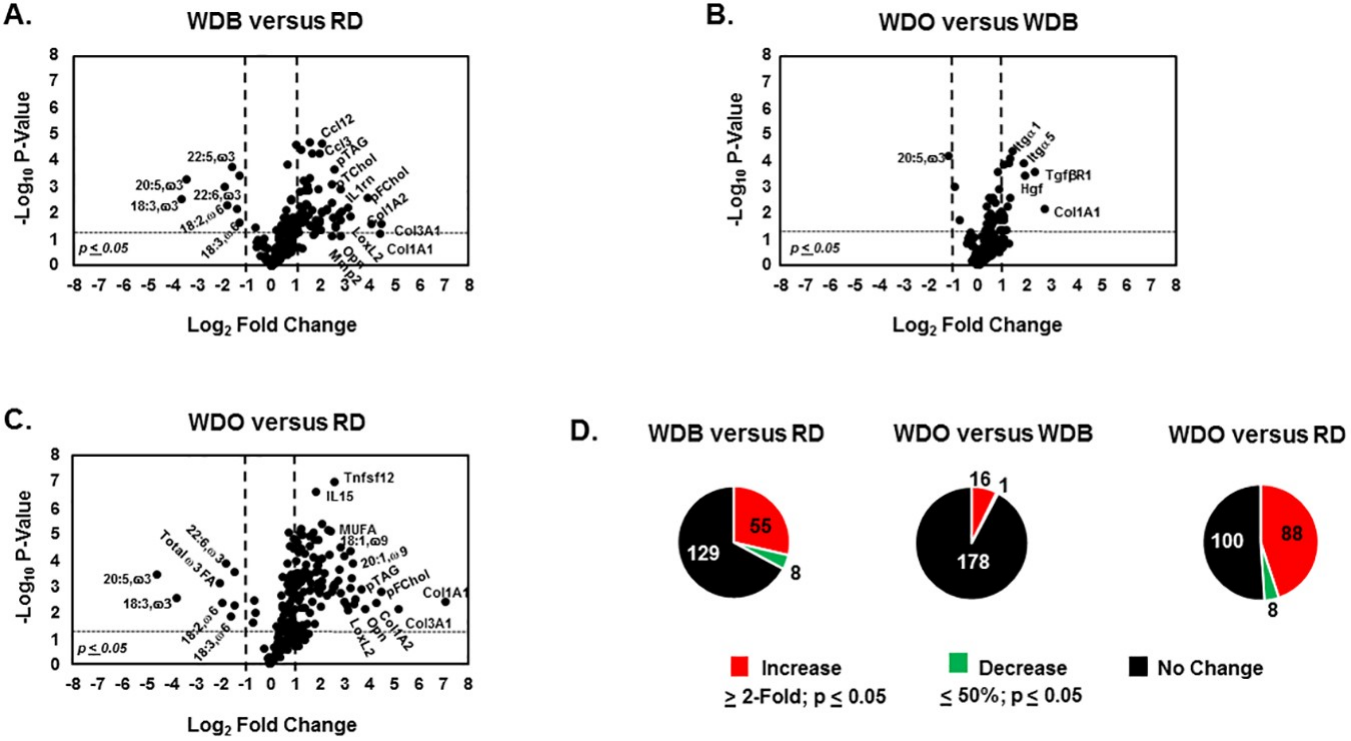
Volcano plots of whole body, plasma and hepatic features in the blockade arm, including the RD, WDB and WDO groups. The analysis examines the overall impact of the WDO and WDD on the progression of WD-induced NASH. Volcano plots were created using the statistical package in MetaboAnalyst 3.0 (http://www.metaboanalyst.ca/MetaboAnalyst/) as well as MS-Excel. The comparisons included WDB versus RD **[A],** WDO versus WDB **[B],** WDO versus RD **[C].** The results were plotted as (–log_10_ p-values) versus fold change (log_2_ fold change). All features included in the heat map (**Fig 4**) were used to construct the volcano plots. The Pie Plots **[D]** represent a summary of the features that increased, decreased or did not change with diet treatment in each comparison.

**Fig 8:**
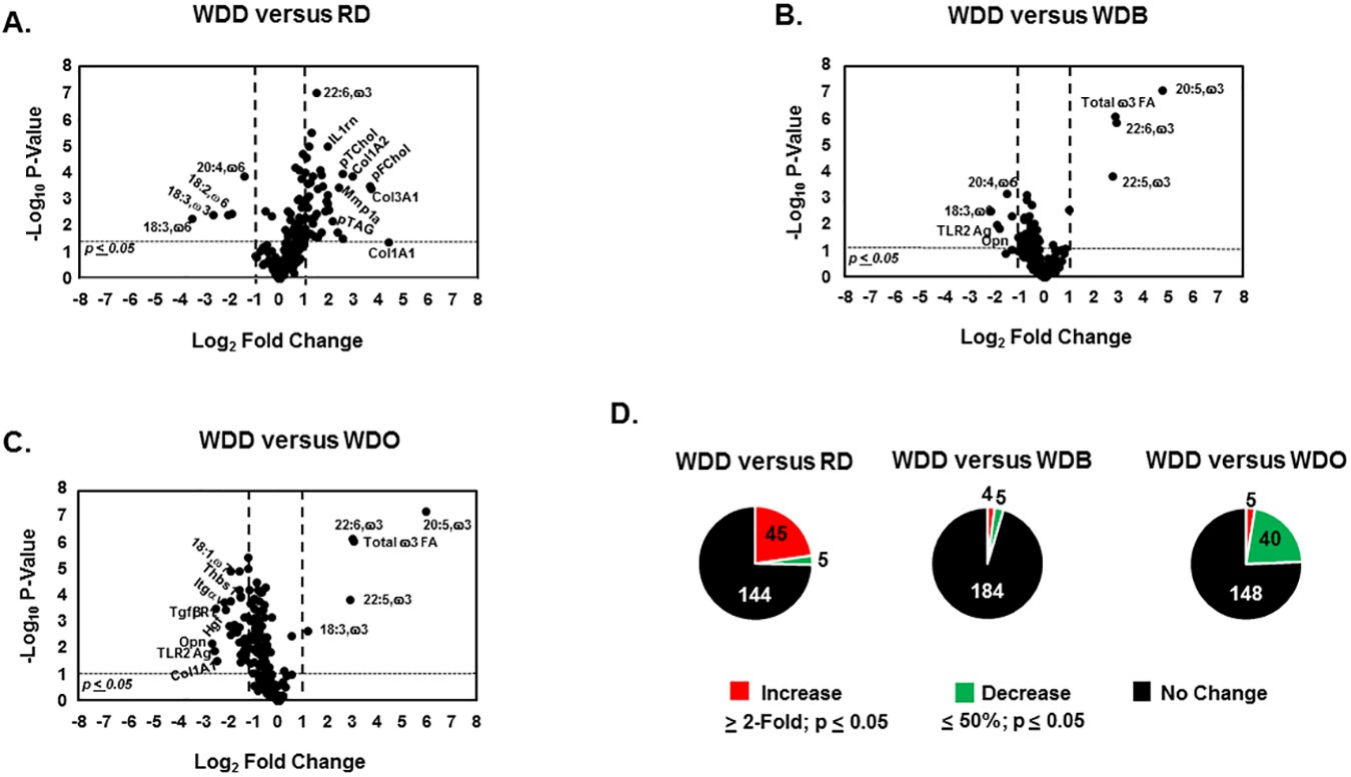
Volcano plots of whole body, plasma and hepatic features in the blockade arm, including the RD, WDD, WDB and WDO Groups. The analysis examines the overall impact of the DHA-mediated blockade of NASH; and includes the WDD versus RD **[A],** WDD versus WDB **[B]** and WDD versus WDO **[C].** As above, the volcano plots were created using the statistical package in MetaboAnalyst 3.0 (http://www.metaboanalyst.ca/MetaboAnalyst/) as well as MS-Excel and used the data represented in the heat map (**Fig 4**). The Pie Plots **[D]** represent a summary of the features that increased, decreased or did not change with diet treatment in each comparison.

To further assess diet effects on disease progression, we used volcano and pie plots (**Figs 7 and 8**). After 22 wks on the WD, the NASH phenotype is characterized by significantly increased plasma triglycerides (pTAG), total and free cholesterol (pTChol, pFChol) and the induction of transcripts encoding multiple collagen subtypes (*Col1A1*, *Col1A2*, *Col3A1*), cytokines and chemokines (*IL1rn*, *Ccl3*, *Ccl12*) (**Fig 7A**). Features that declined significantly in the WDB group included several ω3 and ω6 PUFA. Of the 196 features examined, 55 increased, 8 decreased and 129 remained unchanged by 22 wks on the WD (**Fig 7D**). Sixteen features increased significantly from 22 to 30 wks in the WDO group, including *Col1A1*, *Hgf*, *TfgβR1* and integrins (*Itgα1*, *Itgα5*), while 1 decreased further (20:5,ω3) (**Fig 7B & 7D**). Overall, the WDO group was characterized by a significant increase in 88 features and a significant decrease in 8 features (**S1 Table**). These features included a massive increase in hepatic SFA, MUFA and expression of multiple genes linked to inflammation (*Opn*) and fibrosis (*Col1A1*) and decreases in hepatic ω3 and ω6 PUFA (**Figs 3, 7C & 7D**).

Comparing the WDD and the RD groups showed a significant increase in plasma lipids (cholesterol and triglycerides) and hepatic expression of transcripts associated with inflammation (*IL1rn*) and fibrosis (*Col1A1*, *Col1A2*, *Col3A1*, *Mmp1a*) (**Fig 8A**). However, comparing the WDD and WDB groups revealed no increase in any inflammatory or fibrosis marker (**Fig 8B**). Overall, adding DHA to the WD (WDD group) resulted in the induction of 45 features (**Fig 8D, S2 Table**), whereas adding olive oil to the WD (WDO group) increased 88 features. As such, adding DHA to the WD significantly lowered (~50%) the number of features induced by the WD (**Fig 8D**). In fact, there is no evidence of disease progression in the WDD group. The only features that increased in the WDD group versus WDB group were ω3 PUFA, while 5 features significantly decreased (TLR2 agonist; *Opn;* 18:1,ω7; 18:3,ω6; 20:4,ω6). Thus, DHA addition to the WD blocks NASH progression. All features that increased (≥ 2-fold) or decreased (≥ 50%) significantly (p-value ≤ 0.05) in the comparison of the WDO versus the WDD groups are listed in **S2 Table**.

### DHA effects on NASH remission after switching from a WD to a chow diet: remission arm

**Fig 9:**
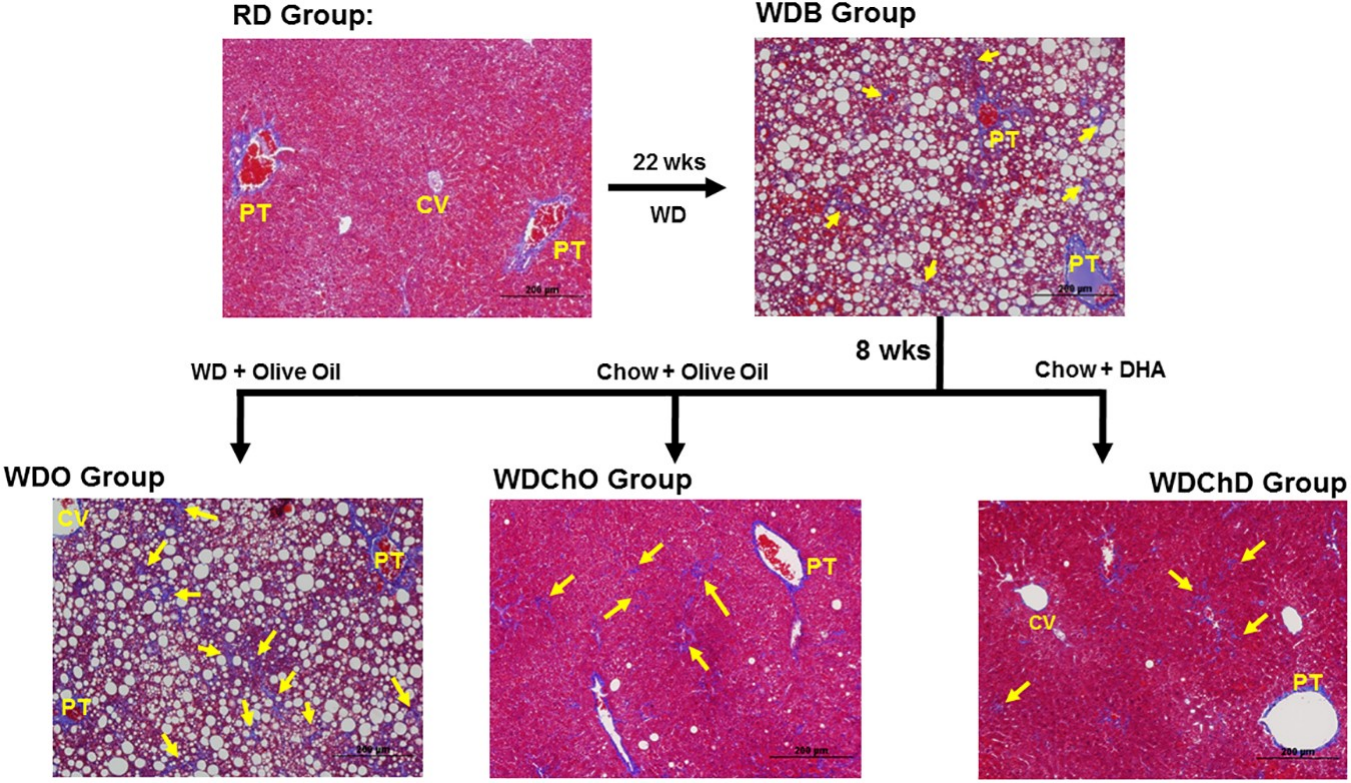
Diet effects on hepatic morphology: Remission arm. Livers sections from the 5 treatment groups (RD, WDB, WDO, WDChO and WDChD) in the Remission Arm were stained with trichrome and photographed at 4x. Lipid droplets appear as white circles, while branching fibrosis appear as blue strands (yellow arrows) in the trichrome stained liver sections. The slides are representative of multiple sections of each liver and all livers in each group. PT, portal track; CV, central vein.

We recently reported that switching mice with NASH from the WD to a chow diet attenuated many, but not all NASH markers in *Ldlr* ^*-/-*^ mice [50]. Herein, we examined the effect of adding DHA to the chow diet on NASH remission (**Fig 1,** Remission Arm). Hepatic histology of mice switched from the WD at 22 wks (WDB) to a chow diet supplemented with either olive oil (WDChO group) or DHA (WDChD group) and euthanized 8 wks later showed nearly a complete loss of steatosis and branching fibrosis **(Fig 9)**. This new study confirms our earlier report that a diet low in fat, cholesterol and sucrose promotes NASH remission [50].

**Fig 10:**
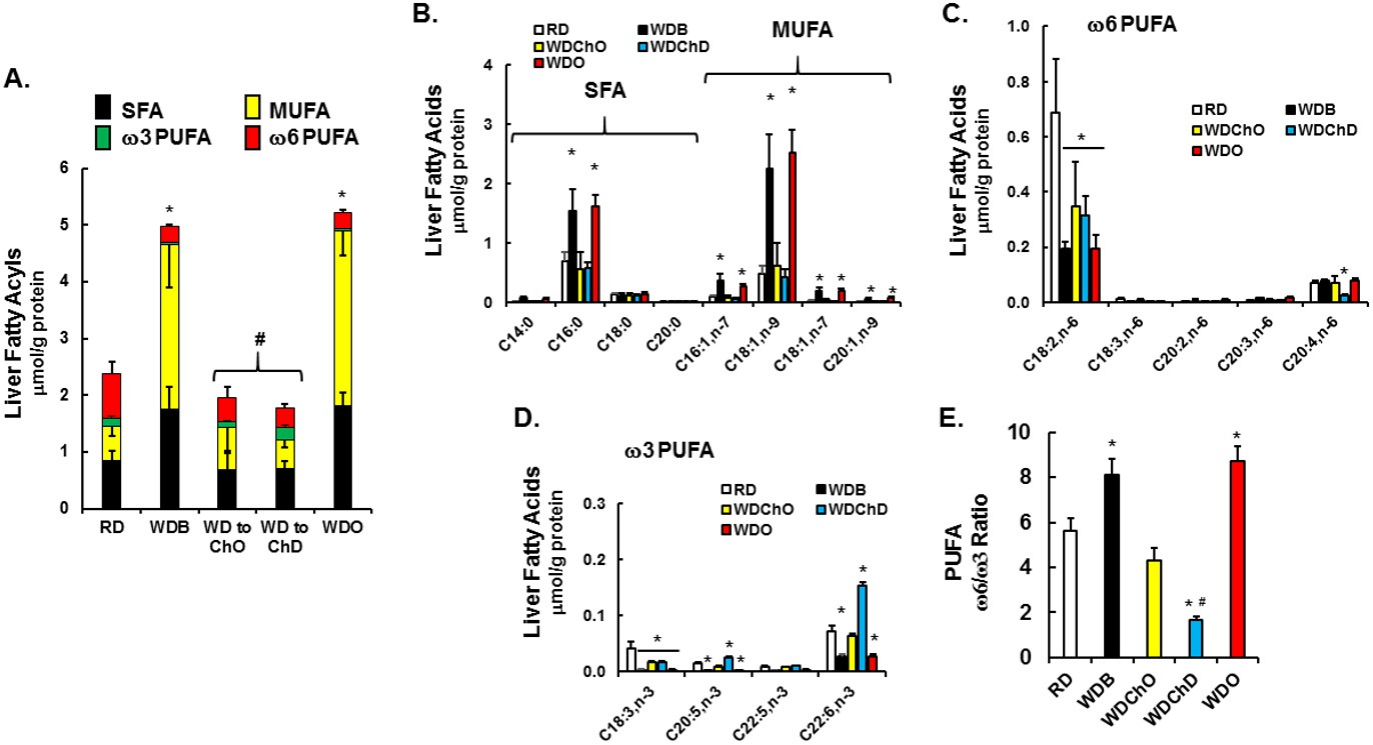
Analysis of hepatic fatty acids: Remission arm. Hepatic fatty acids were quantified as described in Materials and Methods. **A:** The sum of fatty acids in the 4 lipid classes (saturated (SFA), monounsaturated (MUFA), ω3 and ω6 polyunsaturated fatty acids (PUFA) is presented in a stacked histogram. **B-D**: Specific fatty acids in each class are quantified and presented as μmol/mg protein. E: The mole ratio of the sum of ω3 and ω6 PUFA. Mean ± SD with 4–7. *, p<0.05 versus the RD group; #, p<0.05 versus the WDO group using one-way ANOVA.

This remarkable recovery of the liver is associated with a significant decrease in body weight, plasma glucose, lipids (triglycerides, cholesterol), hepatic injury (ALT, AST) and systemic inflammation (TLR2, TLR4 activation) (**Table 2**). Moreover, hepatic weight and fatty acids reveal no significant difference in SFA or MUFA amongst the RD, WDChO or WDChD groups (**Fig 10A & 10B**). The only significant difference between the RD and treatment groups is in ω3 and ω6 PUFA (**Fig 10C**). Hepatic linoleic acid (18:2,ω6) in the WDChO group is ~50% higher when compared to the RD group (**Fig 10C**). Addition of DHA to the chow diet (WDChD group) increased C_20-22_ ω3 PUFA and lowered arachidonic acid levels, leading to a significant reduction in the ω6/ω3 PUFA ratio (**Fig 10D & 10E**).

**Table 2:** Impact of a diet low in fat, sugar and cholesterol on NASH remission: Anthropometric, plasma and liver parameters^1^.

	RD	WDB	WDChO	WDChD	WDO
**Features**					
Body Weight, g	25.0 ± 3.0^a^	38.3 ± 2.3^b^	27.3 ± 2.5^a^	27.3 ± 2.6^a^	41.9 ± 3.1^b^
**Plasma Parameters**					
Glucose, mg/dL	126 ± 66^a^	356 ± 102^b^	226.6 ± 58.9^a^	248 ± 93.5^a^	408 ± 146^b^
Triglycerides, mg/dL	47 ± 28^a^	277 ± 36^b^	124.0 ± 25.5^b^	105.1 ± 64.5^b^	517 ± 218^c^
Cholesterol, mg/dL	143 ± 54^a^	760 ± 118^b^	277.1 ± 63^a^	192.7 ± 49.4^a^	1398 ± 393^c^
ALT, U/L	9.7 ± 2.0^a^	52.0 ± 16.6^b^	6.8 ± 3.9^a^	9.6 ± 3.7^a^	67.1 ± 15.5^b^
AST, U/L	22.1 ± 5.0^a^	57.7 ± 27.3^b,c^	36.1 ± 29.5^a^	50.5 ± 27.8^b^	85.2 ± 17.2^c^
TLR2 Agonist, U/mL	5.1 ± 2.9^a^	9.4 ± 2.9^a^	6.9 ± 3.4^a^	7.2 ± 3.3^a^	14.5 ± 9.7^b^
TLR4 Agonist, U/mL	17.8 ± 7.1^a^	36.5 ± 6.3^b^	21.5 ± 9.2^a^	32.3 ± 10.5^a^	37.5 ± 12.7^b^
**Liver Parameters**					
Liver weight, g	1.15 ± 0.14^a^	2.01 ± 0.3^b^	1.1 ± 0.1^a^	1.2 ± 0.1^a^	2.6 ± 0.7^b^
Liver Weight/BW, g%	4.6 ± 0.2^a^	5.3 ± 0.4^b^	4.0 ± 0.1^a^	4.2 ± 0.1^a^	6.2 ± 1.2^c^
Cholesterol, μg/g protein	27.4 ± 14.5^a^	31.7 ± 9.1^b^	22.1 ± 11.7^a^	19.1 ± 7.0^a^	47.8 ± 14.3^b^
Fatty Acyls, μmol/g protein	2.4 ± 0.6^a^	5.0 ± 1.2^b^	1.9 ± 1.0^a^	1.9 ± 0.3^a^	5.3 ± 0.7^b^

^1^Results are presented as mean ± SD, RD, n = 5; WDB, n = 4; WDChO, n = 6; WDChD, n = 7; WDO, n = 6. Statistical analysis used ANOVA plus Tukey’s HSD to establish statistical significance. Labeled means in a row with superscripts without a common letter differ, P ≤ 0.05.

### Overview of the capacity of DHA to augment chow diet-induced NASH remission

**Fig 11:**
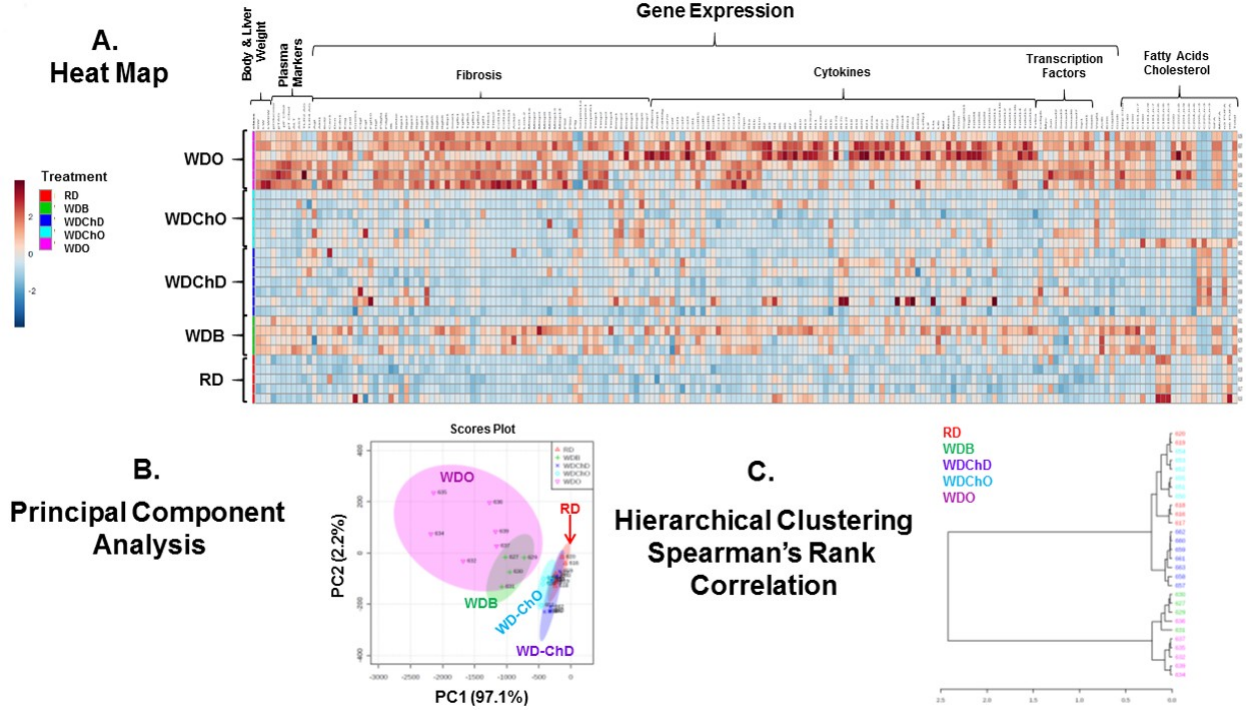
Overview of diet effects on anthropometric, plasma, hepatic gene expression and lipid parameters in the remission arm. As described in **Fig 4**, all anthropometric, plasma, gene expression and hepatic lipid data was assembled into an excel spread sheet for each mouse in each of the 4 groups. The data was analyzed using (http://www.metaboanalyst.ca/MetaboAnalyst/) to create a heat map **[A]** and carry out a principal component analysis **[B]** and hierarchical clustering using Spearman’s ranked correlation **[C].** The heat map is a visualization of the changes in abundance/level of features for each animal identification numbers are listed on the right side of the heat map. The color ranges from deep orange (high abundance or level) to deep blue (low abundance or level); white represents no change.

Using the statistical approach described above, our analysis revealed an impressive capacity of the chow diet to reverse many of the features induced by the WD (**Fig 11**). The PCA plot (**Fig 11B**) shows that the WDChO and WDChD overlap with the RD group; these groups were clearly separated from the WDB and WDO groups. The hierarchical clustering analysis established that although the WDChO and WDChD groups cluster with the RD group, the WDChO appears similar to the RD group, while the WDChD group is a separate subgroup. The factors contributing to this difference likely reflect increased C_20-22_ ω3 PUFA and decrease arachidonic acid in the WDChD group versus the WDChO group (**Figs 10 and 11C**).

### Impact of the chow diet, with and without DHA, on hepatic gene expression in the remission arm

**Fig 12:**
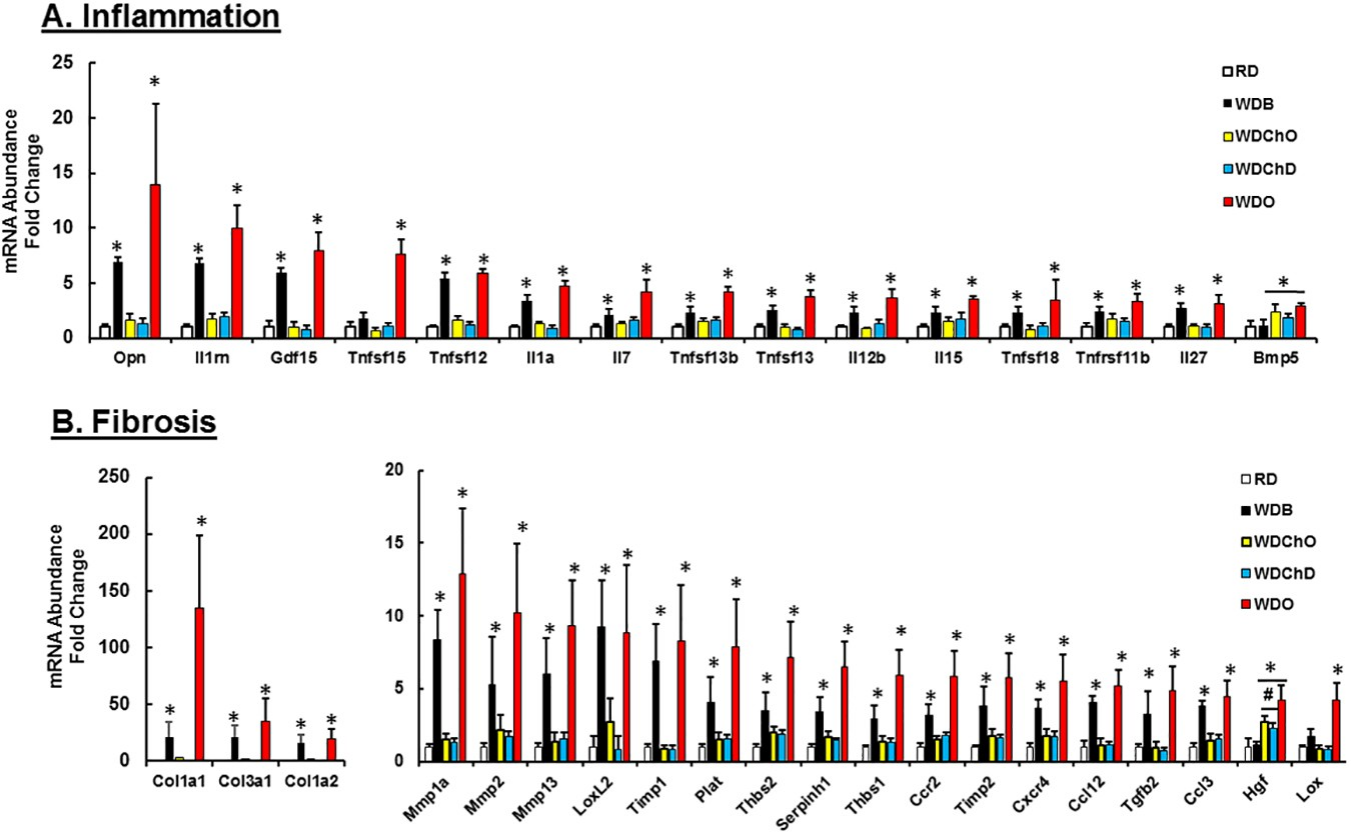
Diet effects on hepatic expression of proteins linked to inflammation and fibrosis in the remission arm. Hepatic transcript abundance of mRNA abundance of transcripts linked to inflammation **[A]** and fibrosis **[B]** was quantified as described in Materials and Methods. Results are represented as mRNA-Fold Change; N = 4–7; mean ± SD with. *, p<0.05 versus the RD group; #, p<0.05 versus the WDO group; one-way ANOVA.

Of the 15 transcripts identified in the cytokine array that were induced ≥ 3-fold by the WD (**Fig 5**), all but one (bone morphogenetic protein 5, ***Bmp5***) were suppressed by returning WD fed mice to the chow diet (WDChO group). In the fibrosis array, nearly all WD-induced transcripts were reduced significantly in the WDChO group. Only hepatic growth factor (***Hgf****)* expression increased after the diet switch from WD to chow (WDChO) (**Fig 12B**).

### DHA augments chow-mediated NASH remission

**Fig 13:**
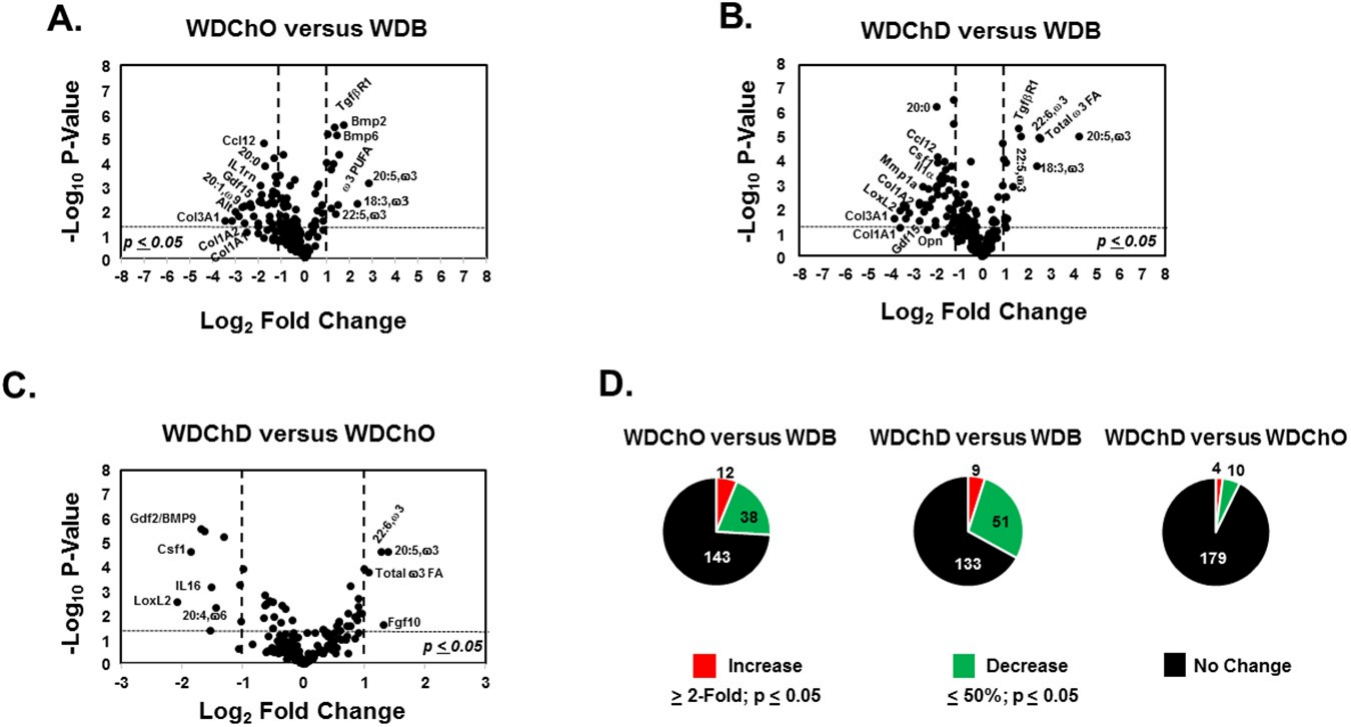
Volcano plots of whole body, plasma and hepatic features in the remission arm, including the WDB, WDChO and WDChD groups. As described above, volcano plots were used to describe the impact of diet on multiple features linked to the NASH. The comparisons examined included: **[A]** WDChO versus WDB; **[B]** WDChD versus WDB and **[C]** WDChD versus WDChO **[D].** The pie plots represent a summary of the features that increased, decreased or did not change with diet treatment.

**Fig 14:**
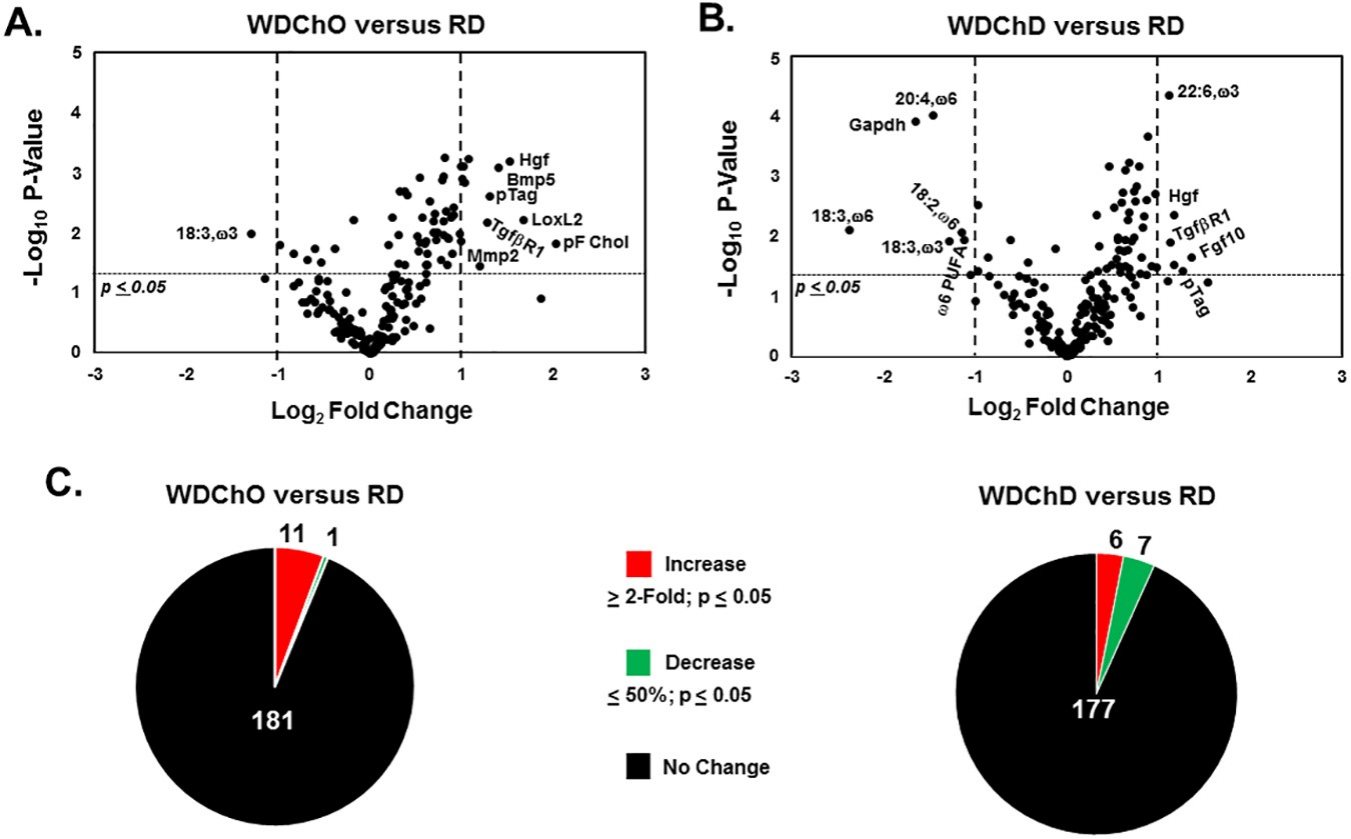
Volcano plots of whole body, plasma and hepatic features in the remission arm, including the RD, WDChO and WDChD groups. As described above, volcano plots were used to describe the capacity of chow diet with and without DHA to return whole body, plasma and hepatic features to levels seen in the RD group. The comparisons included: WDChO versus RD **[A]** and WDChD versus RD **[B]. [C]** The pie plots represent a summary of the features that increased, decreased or did not change with diet treatment.

Volcano and pie plots were used to identify features that were significantly different amongst groups (**Figs 13 & 14**). There is no evidence of disease progression after the mice were switched from the WD to the chow diets (WDChO and WDChD groups), based on histology (**Fig 9**). Comparing the WDChO to the WDB showed that 12 features increased while 38 features decreased (**Fig 13A & 13D**). mRNAs that increased (~2 to 3-fold) include those encoding *TgfβR1*, *Bmp2*, *Bmp6*. The comparison of the WDChD to the WDB group showed an increase in *TgfβR1* expression and hepatic abundance of several ω3 PUFA; overall 9 features were increased in the WDChD group. The WDChD group, however, revealed an attenuation of 51 features, mainly transcripts encoding proteins linked to inflammation and fibrosis (**Fig 13B & 13D**). Comparing the WDChO and WDChD groups showed attenuation of *Gdf2* (also known as *Bmp9*), *Csf1*, *Il16*, *LoxL2*, as well as 20:4,ω6, and increased *Fgf10* expression. These changes were associated with increased hepatic ω3 PUFA content.

Finally, we examined the difference between the WDChO and RD groups; and WDChD and RD groups (**Fig 14**). In the WDChO versus RD comparison, 11 features were increased, while 2 were decreased. Plasma free cholesterol and mRNAs encoding *Hgf*, *Bmp5*, *LoxL2*, *TgfβR1 and Mmp2* increased. In the WDChD versus RD comparison, 6 features were increased, while 7 were decreased. Plasma triglyceride and mRNAs encoding *Hgf*, *TgfβR1*, *& Fgf10* were increased, while only ω6 PUFA were decreased. A comparison of features that significantly differed between the WDChD and WDChO groups (**S3 Table and Fig 13D**) showed that addition of DHA to chow increased hepatic DHA, EPA, total ω3 PUFA and fibroblast growth factor 10 (***Fgf10***), but decreased hepatic transcripts involved in inflammation [lymphotoxin β (***Ltβ***), colony stimulating factor 1 (***Csf1***), interleukin 16 (***IL16***)], fibrosis [lysyl oxidase-like 2 (***LoxL2***), bone morphogenic protein 6 (***Bmp6***)], angiogenesis [vascular endothelial growth factor *α* (***Vegf****α*], metabolism [glyceraldehyde-3 phosphate dehydrogenase (***Gapdh***)] and tissue repair [growth differentiation factor 2 (***Gdf2***)]. The outcome of this analysis established that the addition of DHA to the chow diet offered a significant, but modest improvement, in liver status.

## Discussion

The goal of this study was to evaluate the capacity DHA, alone and in combination with a chow diet to promote weight loss and full remission of WD-induced NASH in a preclinical mouse model. This analysis differs from our previous studies [40, 41, 60] where we tested the capacity of EPA, DHA and EPA + DHA to prevent WD-induced NASH. It also differs from our recent report [50] where we assessed the impact of low fat-low cholesterol diets to reverse NASH.

We used two clinically relevant approaches to promote disease remission. We first examined the capacity of adding DHA to the WD to eliminate all NASH features in mice with pre-existing NASH (**Table 1, Figs 2–8)**. This scenario is clinically relevant since patient compliance to dietary recommendations, i.e., a low fat-low sucrose diet, has historically been poor. We also examined the impact of adding DHA to a diet low in fat, sugar and cholesterol, i.e., chow diet, on NASH remission (**Figs 9–14**). Our studies revealed that DHA supplementation of the WD exerted broad and robust repressive effects on multiple features associated with blood, hepatic lipids and hepatic expression markers of inflammation and fibrosis (**Table 1, Figs 2 & 4**). This treatment approach, however, failed to eliminate several pathological features associated with MetS, including obesity, hyperglycemia or TLR4-associated endotoxinemia. The chow diet, which is low in fat, cholesterol and sucrose, in contrast, significantly attenuated nearly all pathological features associated with MetS and NASH (**Table 2, Figs 9 & 11**). DHA supplementation of the diet low in fat, cholesterol and sugar provided additional benefit over the effects of low fat, cholesterol and sugar diet alone. Overall, these findings support the use of supplemental DHA in the clinical management of patients with NASH.

### Metabolic plasma parameters

Human plasma markers of MetS include hyperglycemia, dyslipidemia (triglycerides and cholesterol) and a low-grade chronic endotoxinemia [76]. *Ldlr*^*-/-*^ mice fed the WD display all of these features, as well as evidence of NASH-associated hepatic injury (ALT & AST) (**Tables 1 & 2**). Switching mice from the WD to the diet low in fat, cholesterol and sugar returned body weight and MetS plasma markers to levels seen in mice fed the reference (RD group; chow) diet (**Tables 1 and 2**), a finding that confirms our previous report [50]. Switching mice from the WD to a WD supplemented with DHA (WDD group) at 22 wks failed to lower blood glucose, ALT or TLR4 agonist levels. While the WDD significantly lowered plasma triglycerides, cholesterol and AST, these plasma markers were not returned to levels seen in the RD group (**Table 1**). Only TLR2 agonist levels returned to levels seen in the RD group.

TLRs play a major role in the onset and progression of NAFLD and fibrosis [77, 78]. In addition to its effects on TLR2 and TLR4 agonist levels, the WD induced hepatic expression of both TLR2 and TLR4, and the ancillary protein CD14 [40]. TLR2 receptors are activated by components from gut-derived gram-positive bacteria, like peptidoglycans and lipoteichoic acid [77], while TLR4 receptors are activated by components from gram-negative gut-derived bacteria, like endotoxin. In addition, both TLR2 and TLR4 activity is regulated by fatty acids. Moreover, saturated fatty acids activate while ω3 PUFA inhibit TLR2 and TLR4 function [79]. The WD induces endotoxinemia [41] and severe dyslipidemia (**Table 1**). Correlation analyses establish a strong association between plasma TLR2 agonist levels and hepatic features linked to inflammation (C-X-C chemokine 4 receptor, interleukin 1*α*) and fibrosis (*Timp1*, *2*, *3; Mmp 1a & 14; TgfβR1 & TgfβR2*) (**S4 Table)**. DHA attenuates WD-mediated induction of these features, while the chow diet returns expression levels of these transcripts to levels seen in the RD group. TLR effects on hepatic gene expression are mediated through interferon regulatory factors (IRF-3 & 7), NFκB, Jnk and p38 Mapk. Feeding mice the WD increases NFκB-p50 and NFκB-p65 in hepatic nuclei, while DHA suppresses WD-induced NFκB-p50 nuclear content [40]. The impact of DHA on IRF-3 &7, Jnk and p38 MapK in this model has not been examined. Equally unclear is whether DHA effects on plasma TLR2 agonist levels are linked to effects on blood levels of gut-derived bacterial components or plasma lipids.

### Hepatic inflammation and cytokines

A key pathological feature of NASH is hepatic inflammation, a process that involves infiltration of the liver by monocytes, macrophage and T-cells; and the production of chemokines, cytokines and factors contributing to oxidative stress. After 22 and 30 wks on the WD, 11 and 14 transcripts were induced, respectively, at least 3-fold (**Fig 5**). The most abundant hepatic transcript induced by the WD was *Opn*, a secreted phosphoprotein appearing in plasma. Immunoblot analysis established that changes in hepatic Opn mRNA abundance correlated well with changes in hepatic OPN protein abundance (**Fig 6**). Opn expression is regulated by the Notch, Hedgehog [80, 81] and vitamin D receptor-Runx2 pathways [82]. Opn is expressed in multiple cell types and is upregulated in certain cancers [83, 84]. Opn activates cells by binding integrins (**Itgα5 & Itgβ3**) and **CD44**, a ubiquitously expressed receptor for hyaluronic acid (a soluble marker of fibrosis). CD44 is involved in cell growth and migration [85, 86]. Opn interaction with these cell surface proteins has been implicated in alcoholic liver disease progression and it has served as a marker of hepatic fibrosis progression [73] and HCC [87]. Correlation analyses establish a strong association between Opn expression and fibrosis (**S5 and S6 Tables**). Recent reports indicate that Opn regulates HMGB1 expression [88], and together with Opn, acts in a paracrine fashion as a downstream “alarmin” driving Col1A1 synthesis in hepatic stellate cells. Adding DHA to the WD (**Fig 6**) or switching from the WD to chow diet (**Fig 12**) essentially returned Opn transcript abundance to levels seen in the RD group.

Many of the cytokine-linked transcripts that responded to changes in diet are expressed at low levels in liver (**S1 Fig**). The cytokine array identified several interleukins (*Il1α*, *Il1rn*, *Il5*, *Il7*, *Il12b*, *Il27*) and members of the tumor necrosis factor (TNF) superfamily (*Tnfsf 12*, *13b*, *15*, *and 18*) as well as a growth differentiation factor (*Gdf15*) and a bone morphogenetic protein (*Bmp5*) as targets for diet control. These transcripts were well-induced by the WD and suppressed by DHA addition to the WD (**Fig 5**) or by switching from the WD to the chow diet at 22 wks (**Fig 12**). Interleukins are produced by macrophages, monocytes, T-cells and endothelial cells; they regulate immune response as well as cell survival and proliferation [89, 90]. Interleukins are classically considered “pro-inflammatory” [90–95], while DHA generally functions in an “anti-inflammatory” capacity [96].

*Gdf15* is induced in humans with liver cirrhosis and hepatocellular carcinoma [97], while several BMPs, which are members of the TGFβ superfamily [98], are induced in NASH, cirrhosis and HCC. *Gdf15* is induced after 22 wks on the WD, but *Bmp5* is significantly induced at 30 wks on the WD. While addition of DHA to the WD at 22 wks blocks *Bmp5* induction at 30 wks (**Fig 5**), switching from the WD at 22 wks to the chow diet, without or with DHA does not block Bmp5 induction (**Fig 12**). Since DHA significantly attenuates *Gdf15* and Bmp5 expression, DHA may prevent NASH progression to HCC. Future studies will establish if diet-induced changes in these transcripts are associated with significant changes in the corresponding proteins.

### Hepatic fibrosis

Diet-induced hepatic inflammation promotes liver injury leading to tissue repair and fibrosis. While our previous reports dealt with prevention, this report focused on stopping disease progression and promoting disease remission. Clear evidence of fibrosis progression is seen in the significant increases in *Col1A1*, *Thbs1 & 2*, *Hgf* and *Lox* expression at 30 versus 22 wks on the WD (**Fig 5**). Col1A1 expression shows a strong correlation with multiple transcripts linked to fibrosis (**S7 and S8 Tables**), a finding that reveals coordinate control. TGFβ is a major regulator of hepatic fibrosis [61] and a target of DHA suppression [60]. Lox is involved in collagen cross-linking [60] and hepatic growth factor (*Hgf*) plays a role in repair of hepatic damage [99].

While our previous studies described the capacity of DHA to prevent WD-induced hepatic fibrosis [40, 60], these findings makes clear that inclusion of DHA in the diet of WD fed animals with well-established NASH and fibrosis blocks fibrosis progression and may even lower hepatic fibrosis (**Figs 2 & 7**). At least one mechanism for WD induced and DHA attenuated fibrosis is through suppression of hepatic nuclear content phospho-Smad3, a key mediator of TGFβ signaling [60].

The WD appears to coordinately induced the expression of multiple proteins linked to ECM deposition in the liver (*Col1A1*, *Timp1 & 2*, *Lox/LoxL2*), while also inducing expression of enzymes linked to basement membrane remodeling and fibrosis removal, such as matrix metalloproteases (*Mmp1a*, *2 & 13*) (**S7 and S8 Tables**). DHA attenuates WD-mediated induction of these proteins in a prevention study [60] and blocks further induction of these transcripts in a remission study (**Fig 5A**). The immune system plays multiple roles in NASH; it is involved in both the onset and progression of NASH as well as its remission. Products of macrophage are required for the resolution of NASH since these cells are the source of enzymes that remove collagen, e.g., matrix metalloprotease [63, 100]. Due to the limitations of the experimental design, it is unclear if the DHA-mediated suppression of *Mmp1a*, *2 and 13a* is due to DHA suppression of expression of these enzymes in innate immune cells involved in resolution of inflammation or due to an overall suppression of inflammation. Well-designed time course studies coupled with analysis of DHA effects on innate immune cells and the corresponding Timp and Mmp proteins will be required to resolve this issue.

### Hepatic PUFA

Arendt, et al [42] recently reported a decline in hepatic ω3 and ω6 PUFA in the transition from simple steatosis to NASH in humans. Changes in mouse and human liver PUFA content are associated with changes in expression of *Fads1*, *Fads2 & Elovl5* expression, key enzymes involved in PUFA synthesis [40, 60]. The suppression of PUFA production is also seen in wild type mice fed high fat diet; and high fat diets alter FoxO1 and mTorc2 signaling and ATGL expression to reduce triglyceride catabolism [36, 101, 102]. Thus, our studies support recent human studies showing that high fat diets negatively impact PUFA metabolism and liver health.

Addition of DHA to the WD, at 2% total calories, significantly increased hepatic DHA, DPA and EPA content and lowered MUFA and arachidonic acid content as well as significantly lowering the ω6/ω3 PURA ratio (**Fig 3**). MUFA content decreases because of suppression of SCD1 expression, while arachidonic acid content decreased because of suppressed Fads1, Fads2 & Elovl5 expression [40]. EPA and DPA increase as a result of DHA retroconversion [103].

Finally, DHA induced hepatic *Fgf10*, a growth factor involved in hepatic embryonic development [104]. While a role for Fgf10 has not been described in the context of NASH remission, its role in early organogenesis and development and patterning between the liver and pancreas has been described [105]. As noted above, we have uncovered gene expression markers of advanced NASH and HCC, i.e., *Gdf15 and Bmp5*. What role Fgf10 plays in NASH remission or its progression to HCC will require further study.

## Conclusions

We have established that using DHA as a dietary supplement for the treatment of pre-established WD-induced NASH in *Ldlr*^*-/-*^ mice blocked disease progression, decreased plasma triglycerides and decreased existing hepatosteatosis, inflammation- and fibrosis-associated gene expression markers and histological evidence of hepatic fibrosis. Supplemental DHA, however, did not lower hepatic injury as evidenced by no significant reduction of ALT levels. We assessed the reversibility of NASH by returning mice with WD-induced NASH to a low-fat low-cholesterol chow diet without or with supplemental DHA for 8 weeks. This diet returned NASH livers to livers with minimal evidence of hepatic steatosis, inflammation, fibrosis or liver injury within 8 wks. This treatment, while ideal for clinical use, is likely not sustainable in NASH patients due to poor compliance to dietary therapy. As such, our studies with the *Ldlr*^*-/-*^ mouse suggest that adding DHA (at ~2% total energy) to a patient’s regular diet will block disease progression, which is a key goal in disease management.

## Supporting information

S1 Table Features which differ significantly between the WDO versus RD groups. (DOCX)

S2 Table Features which differ significantly between the WDD versus WDO groups. (DOCX)

S3 Table Features which differ significantly between the WDChO versus WDChD groups. (DOCX)

S4 Table Top 20 correlations between plasma TLR2 agonists and hepatic features: Blockage and Remission Arms1. (DOCX)

S5 Table Top 20 Features Correlating with Hepatic Osteopontin Expression: Blockage Arm. (DOCX)

S6 Table Top 20 features correlating with hepatic Osteopontin expression: Remission Arm. (DOCX)

S7 Table Top 20 Features correlating with hepatic collagen 1A1 expression: Blockage Arm. (DOCX)

S8 Table Top 20 Features correlating with hepatic collagen 1A1 expression: Remission Arm. (DOCX)

S1 Fig **Relative abundance of hepatic transcripts linked to inflammation [A] and fibrosis [B]:** Hepatic mRNA abundance was quantified using qRTPCR arrays as described in Materials and Methods. The reference gene was Hsp90. Results are represented as mRNA Abundance, Relative to Hsp90 (reference gene) for the RD group only. N = 5; mean ± SD.
(TIF)

S2 Fig Diet effects on hepatic transcripts encoding Mcp1, TLR4, TNFα Diet effNox2. **A-E:** Hepatic mRNA abundance was quantified as described in Materials and Methods using in-house PCR primers. Results are represented as mRNA Abundance-Fold Change; N = 4–7; mean ± SD; *, p<0.05 versus the RD group; #, p<0.05 versus the WDO group; one-way ANOVA. **F:** Hepatic mRNA abundance was quantified using qRTPCR arrays as described in Materials and Methods. The reference gene was cyclophilin. Results are represented as mRNA Abundance, Relative to Cyclophilin (reference gene) for the RD group only. N = 5; mean ± SD.
(TIF)
