# Supplementary material for: Docosahexaenoic acid blocks progression of western diet-induced nonalcoholic steatohepatitis in obese Ldlr-/- mice
Source: PLoS One. 2017 Apr 19;12(4):e0173376. doi: 10.1371/journal.pone.0173376 (PMC5396882; doi:10.1371/journal.pone.0173376)
Supplement: S1 Table — (DOCX) [file pone.0173376.s001.docx]

**S1 Table:**

**Features that differ significantly between the WDO versus RD groups^1^.**

| **Feature** |  | **Fold Change** | **p-Value** |  | **Feature** |  | **Fold Change** | **p-Value** |
| --- | --- | --- | --- | --- | --- | --- | --- | --- |
| Col1A1 | Collagen 1A1 | 134.64 | 0.0037 |  | Tnfrsf11b | TNF superfamily 11b | 3.34 | 7.59E-05 |
| Col3A1 | Collagen 3A1 | 35.74 | 0.0075 |  | Tgfβ1 | Transforming growth factor β1 | 3.32 | 8.54E-06 |
| pF Chol | Plasma free cholesterol | 22.25 | 0.0016 |  | pGlucose | Plasma glucose | 3.23 | 0.0036 |
| Col1A2 | Collagen 1A2 | 19.38 | 0.0043 |  | Il27 | Interleukin 27 | 3.12 | 0.0007 |
| Opn | Osteopontin | 13.94 | 0.0077 |  | Itgαv | Integrin α5 | 3.02 | 0.0003 |
| Mmp1A | Matrix metalloprotease 1A | 12.87 | 0.0013 |  | Dcn | Decorin | 2.94 | 0.0015 |
| pTAG | Plasma triglyceride | 10.89 | 0.0031 |  | Acta2 | Smooth muscle actin | 2.94 | 0.0104 |
| Mmp2 | Matrix metalloprotease 2 | 10.20 | 0.0050 |  | Bmp5 | Bone morphogenetic protein 5 | 2.85 | 0.0004 |
| Il1rn | Interleukin 1 receptor antagonist | 9.94 | 0.0001 |  | Tnfsf8 | TNF superfamily 8 | 2.74 | 0.0268 |
| pT Chol | Plasma total cholesterol | 9.73 | 0.0005 |  | Tgfβ3 | Transforming growth factor β3 | 2.73 | 0.0001 |
| Mmp13 | Matrix metalloprotease 13 | 9.35 | 0.0011 |  | Itgβ8 | Integrin β8 | 2.70 | 0.0005 |
| 20:1,ω9 | Gondolic acid | 9.27 | 4.33E-05 |  | Il18 | Interleukin 18 | 2.70 | 0.0003 |
| LoxL2 | Lysyl oxidase-like 2 | 8.86 | 0.0080 |  | 20:0 | Arachidic acid | 2.64 | 0.0065 |
| Timp1 | Tissue inhibitor protease 1 | 8.29 | 0.0056 |  | Stat1 | Signal transducer & activator of transcription | 2.62 | 0.0002 |
| Gdf15 | Growth differentiation factor 15 | 7.94 | 6.49E-05 |  | Itgα1 | Integrin α1 | 2.59 | 5.22E-05 |
| Plat | Plasminogen activator | 7.90 | 0.0036 |  | Tnfsf9 | TNF superfamily 9 | 2.48 | 0.0015 |
| Thbs2 | Thrombospondin 2 | 7.11 | 0.0019 |  | Tnfsf15 | TNF superfamily 15 | 2.45 | 0.0318 |
| 18:1,ω7 | Cis-vaccenic acid | 7.05 | 3.22E-05 |  | Mmp8 | Matrix metalloprotease 8 | 2.43 | 1.06E-05 |
| ALT | Alanine aminotransferase | 6.95 | 0.0002 |  | Csf1 | Colony stimulating factor 1 | 2.42 | 0.0005 |
| Serpinh1 | Serpin peptidase H1 | 6.50 | 0.0006 |  | 20:3,ω6 | Homo-γ-linolenic acid | 2.35 | 0.0063 |
| Thbs1 | Thrombospondin 2 | 5.91 | 0.0011 |  | Fasl | FAS ligand | 2.33 | 0.0007 |
| Tnfsf12 | TNF superfamily 12 | 5.89 | 9.83E-08 |  | Il23a | Interleukin 23a | 2.32 | 3.51E-05 |
| Ccr2 | Chemokine receptor 2 | 5.88 | 0.0008 |  | Itgβ6 | Integrin β6 | 2.30 | 6.04E-06 |
| Timp2 | Tissue inhibitor protease 2 | 5.75 | 0.0010 |  | 16:0 | Palmitic acid | 2.29 | 8.03E-06 |
| Cxcr4 | C-X-C chemokine receptor 4 | 5.50 | 0.0016 |  | Il24 | Interleukin 24 | 2.28 | 0.0383 |
| 18:1,ω9 | Oleic acid | 5.30 | 7.23E-06 |  | LW | Liver weight | 2.27 | 0.0027 |
| Ccl12 | Chemokine ligand 12 | 5.18 | 5.82E-05 |  | Lif | Leukemia inhibitor factor | 2.21 | 0.0344 |
| MUFA | Monounsaturated fatty acids | 5.08 | 6.49E-06 |  | Smad6 | Mothers against decapentaplegic homolog 6 | 2.21 | 0.0022 |
| Tgfβ2 | Transforming growth factor β2 | 4.85 | 0.0022 |  | Total FA | Total saponifiable fatty acids | 2.20 | 4.851E-05 |
| Ccl3 | Chemokine ligand 3 | 4.46 | 0.0003 |  | Ltβ | Lymphotoxin β | 2.20 | 0.0003 |
| Hgf | Hepatic growth factor | 4.26 | 8.66E-05 |  | Snai1 | Snail family zinc finger 1 | 2.17 | 0.0005 |
| Lox | Lysyl oxidase | 4.24 | 0.0010 |  | TLR4 AG | Toll like receptor 4 agonist | 2.14 | 0.0036 |
| Il7 | Interleukin 7 | 4.23 | 0.0006 |  | SFA | Saturated fatty acids | 2.13 | 2.08E-05 |
| Tnfsf13b | TNF superfamily 13b | 4.15 | 3.94E-06 |  | Smad3 | Mothers against decapentaplegic homolog 3 | 2.13 | 0.0005 |
| Serpine1 | Serpin peptidase E1 | 4.03 | 0.0057 |  | Itgβ5 | Integrin β5 | 2.13 | 0.0007 |
| Ltbp1 | Latent transforming growth factor binding protein 1 | 3.92 | 0.0017 |  | Myc | Myelocytomatosis oncogene | 2.12 | 0.0009 |
| AST | Aspartate aminotransferase | 3.85 | 0.0001 |  | Smad7 | Mothers against decapentaplegic homolog 7 | 2.07 | 0.0001 |
| Tnfsf13 | TNF superfamily 13 | 3.80 | 1.71E-05 |  | Fgf10 | Fibroblast growth factor 10 | 2.04 | 0.0319 |
| Pdgf-B | Platelet derived growth factor B | 3.79 | 0.0002 |  | Edn1 | Endothelin 1 | 2.03 | 0.0003 |
| Bcl2 | B-cell lymphoma 2 gene | 3.77 | 8.81E-05 |  | ω6 PUFA | ω6 Polyunsaturated fatty acids | 0.37 | 0.0055 |
| 14:0 | Myristic acid | 3.75 | 0.0008 |  | 22:6,ω3 | Docosahexaenoic acid | 0.36 | 0.0003 |
| Tgfβr2 | TGFβ receptor 2 | 3.72 | 0.0011 |  | 18:3,ω6 | γ-Linolenic acid | 0.33 | 0.0149 |
| Il12b | Interleukin 12b | 3.70 | 0.0002 |  | 22:5,ω3 | Docosapentaenoic acid | 0.28 | 0.0001 |
| Timp3 | Tissue inhibitor protease 3 | 3.62 | 0.0013 |  | 18:2,ω6 | Linoleic acid | 0.26 | 0.0041 |
| Tgfβr1 | TGFβ receptor 1 | 3.59 | 0.0003 |  | ω3 PUFA | ω3 Polyunsaturated fatty acids | 0.23 | 0.0007 |
| Il15 | Interleukin 15 | 3.52 | 2.34E-07 |  | 18:3,ω3 | α-Linolenic acid | 0.07 | 0.0027 |
| Tnfsf18 | TNF superfamily 18 | 3.41 | 0.0263 |  | 20:5,ω3 | Eicosapentaenoic acid | 0.04 | 0.0004 |
| Il1α | Interleukin 1α | 3.37 | 1.57E-05 |  |  |  |  |  |

^1^All data used to construct the heat map in Fig 4 was used for statistical analysis using the MetaboAnalyst 3.0 statistical package. The table lists all features that differ significantly, p < 0.05 between the WDO and WDD groups as determined by ANOVA-Tukey HSD.
