# Supplementary material for: Docosahexaenoic acid blocks progression of western diet-induced nonalcoholic steatohepatitis in obese Ldlr-/- mice
Source: PLoS One. 2017 Apr 19;12(4):e0173376. doi: 10.1371/journal.pone.0173376 (PMC5396882; doi:10.1371/journal.pone.0173376)
Supplement: S2 Table — (DOCX) [file pone.0173376.s002.docx]

|  |  |  |  |
| --- | --- | --- | --- |
|  |  |  |  |
|  |  |  |  |

|  |
| --- |

**S2 Table:**

**Features that differ significantly between the WDD versus WDO groups^1^.**

| **Feature** |  | **Fold Change** | **T-test** |  | **Feature** |  | **Fold Change** | **T-Test** |
| --- | --- | --- | --- | --- | --- | --- | --- | --- |
| 20:5,ω3 | Eicosapentaenoic Acid | 60.98 | 5.7 x10^-8^ |  | Plat | Plasminogen activator | 0.38 | 0.0138 |
| ω3 PUFA | ω3 Polyunsaturated fatty Acids | 8.38 | 8.1 x10-^7^ |  | 20:1,ω9 | Gondolic Acid | 0.35 | 9.5 x 10^-5^ |
| 22:6,ω3 | Docosahexaenoic Acid | 7.90 | 6.5 x 10^-7^ |  | Smad3 | Mothers against decapentaplegic homolog 3 | 0.35 | 0.0001 |
| 22:5,ω3 | Docosapentaenoic Acid | 7.54 | 0.0001 |  | Mmp2 | Matrix metalloprotease 2 | 0.35 | 0.0175 |
| 18:3,ω3 | α-Linolenic Acid | 2.26 | 0.0020 |  | Col3A1 | Collagen 3A1 | 0.35 | 0.0335 |
| Itgβ8 | Integrin β8 | 0.50 | 0.0045 |  | 20:4,ω6 | Arachidonic acid | 0.35 | 1.1 x 10^-5^ |
| Inhβa | Inhibin βa | 0.49 | 0.0002 |  | Serpinh1 | Serpin peptidase inhibitor H1 | 0.34 | 0.0016 |
| Il7 | Interleukin 7 | 0.47 | 0.0034 |  | Thbs2 | Thrombospondin 2 | 0.34 | 0.0055 |
| Tnfsf13b | TNF superfamily 13b | 0.47 | 0.0002 |  | Itgα1 | Integrin α1 | 0.34 | 5.9 x 10^-5^ |
| Timp1 | Tissue inhibitor metaloprotease1 | 0.47 | 0.0360 |  | Thbs1 | Thrombospondin 1 | 0.31 | 0.0024 |
| Cxcr4 | C-X-C Chemokine receptor 4 | 0.46 | 0.0095 |  | Lox | Lysyl oxidase | 0.30 | 0.0012 |
| Pdgf-B | Platelet derive growth factor B | 0.46 | 0.0009 |  | TGFβR2 | TGFβ receptor-2 | 0.29 | 0.0017 |
| Tnfsf13 | TNF superfamily 13 | 0.46 | 6.0 x 10^-5^ |  | 18:3,ω6 | γ-Linolenic acid | 0.27 | 0.0001 |
| AST | Aspartate aminotransferase | 0.44 | 0.0006 |  | 18:1,ω7 | Cis-vaccenic acid | 0.27 | 1.1 x 10^-5^ |
| Bmp5 | Bone morphogenetic protein 5 | 0.44 | 9.4 x 10^-6^ |  | TGFβ2 | TGFβ2 | 0.26 | 0.0028 |
| Mmp13 | Matrix metalloprotease 13 | 0.41 | 0.0056 |  | Timp3 | Tissue inhibitor metalloprotease 3 | 0.26 | 0.0014 |
| Gdf15 | Growth differentiation factor 15 | 0.41 | 0.0006 |  | HGF | Hepatic growth factor | 0.24 | 0.0003 |
| Mmp1a | Matrix metalloprotease 1a | 0.41 | 0.0078 |  | Itgα5 | Integrin α5 | 0.22 | 0.0002 |
| pTAG | Plasma triglyceride | 0.41 | 0.0167 |  | TLR2 Agonist | Toll like receptor 2 agonist | 0.18 | 0.0301 |
| Col1A2 | Collagen 1A2 | 0.40 | 0.0257 |  | TGFβR1 | TGFβ receptor-1 | 0.18 | 0.0003 |
| Timp2 | Tissue inhibitor metalloprotease 2 | 0.40 | 0.0039 |  | Opn | Osteopontin | 0.17 | 0.0117 |
| Il1rn | Interleukin 1 Receptor antagonist | 0.39 | 0.0007 |  | Col1A1 | Collagen 1A1 | 0.16 | 0.0063 |
| Jun | Jun transcription factor | 0.39 | 0.0108 |  |  |  |  |  |

^1^All data used to construct the heat map in Fig 4 was used for statistical analysis using the MetaboAnalyst 3.0 statistical package. The table lists all features that differ significantly, p < 0.05 between the WDD and WDO groups as determined by ANOVA-Tukey HSD.
