# Supplementary material for: Docosahexaenoic acid blocks progression of western diet-induced nonalcoholic steatohepatitis in obese Ldlr-/- mice
Source: PLoS One. 2017 Apr 19;12(4):e0173376. doi: 10.1371/journal.pone.0173376 (PMC5396882; doi:10.1371/journal.pone.0173376)
Supplement: S3 Table — (DOCX) [file pone.0173376.s003.docx]

**S3 Table:**

**Features that differ significantly between the WDChD versus WDChO groups^1^.**

| **Feature** |  | **Fold Change** | ***p-value*** |
| --- | --- | --- | --- |
|  |  |  |  |
| 20:5,ω3 | Eicosapentaenoic acid | 2.61 | 2.25 x 10^-5^ |
| Fgf10 | Fibroblast growth factor 10 | 2.47 | 0.024 |
| 22:6,ω3 | Docosahexaenoic acid | 2.40 | 2.33 x 10^-5^ |
| ω3 PUFA | ω3 Polyunsaturated fatty acids | 2.08 | 0.00016 |
| Ltβ | Lymphotoxin β | 0.48 | 0.018 |
| Bmp6 | Bone morphogenetic protein 6 | 0.47 | 0.00053 |
| Vegfα | Vascular endothelial growth factor α | 0.40 | 5.8 x 10^-6^ |
| 20:4,ω6 | Arachidonic acid | 0.35 | 0.0046 |
| Il16 | Interleukin 16 | 0.34 | 0.00069 |
| 18:3,ω6 | γ-Linolenic acid | 0.34 | 0.045 |
| Gapdh | Glyceraldehyde 3-phosphate dehydrogenase | 0.32 | 3.21 x 10^-6^ |
| Gdf2 | Growth differentiation factor 2 (BMP9) | 0.31 | 2.5 x 10^-6^ |
| Csf1 | Colony stimulating factor 1 | 0.27 | 2.36 x 10^-5^ |
| LoxL2 | Lysyl oxidase-like 2 | 0.23 | 0.0026 |
|  |  |  |  |

^1^All data used to construct the heat map in Fig 11 was used for statistical analysis using the MetaboAnalyst 3.0 statistical package. The table lists all features that differ significantly, p < 0.05, between the WDChO and WDChD groups as determined by ANOVA-Tukey HSD.
