# Supplementary material for: Docosahexaenoic acid blocks progression of western diet-induced nonalcoholic steatohepatitis in obese Ldlr-/- mice
Source: PLoS One. 2017 Apr 19;12(4):e0173376. doi: 10.1371/journal.pone.0173376 (PMC5396882; doi:10.1371/journal.pone.0173376)
Supplement: S4 Table — (DOCX) [file pone.0173376.s004.docx]

**S4 Table:**

**Top 20 correlations between plasma TLR2 agonists and hepatic features: Blockage and Remission Arms^1^**

| **Feature** |  | **Correlation *r-value*** | ***p-value*** |
| --- | --- | --- | --- |
|  |  |  |  |
| Cxcr4 | C-X-C chemokine receptor type 4 | 0.66 | 1.9 x 10-5 |
| Timp3 | Tissue inhibitor metalloprotease 3 | 0.66 | 1.9 x 10-5 |
| Mmp14 | Matrix metalloprotease 14 | 0.65 | 3.5 x 10-5 |
| Tgfβr2 | Transforming growth factor β receptor 2 | 0.64 | 4.9 x 10-5 |
| Stat6 | Signal transducer & activator of transcription 6 | 0.63 | 6.2 x 10-5 |
| Sp1 | Specificity protein 1 | 0.63 | 7.1 x 10-5 |
| Tgfβi1 | TGFβ induced homeobox protein 1 | 0.62 | 9.2 x 10-5 |
| Mmp1α | Matrix metalloprotease 1α | 0.61 | 0.00011 |
| LW %BW | Liver weight % body weight | 0.61 | 0.00011 |
| Timp2 | Tissue inhibitor metalloprotease 2 | 0.61 | 0.00011 |
| Tgfβr1 | Transforming growth factor β receptor 1 | 0.61 | 0.00015 |
| Hgf | Hepatic growth factor | 0.60 | 0.00017 |
| Tnfsf13 | TNF superfamily 13 | 0.59 | 0.00023 |
| Itgα1 | Integrin α1 | 0.58 | 0.00030 |
| Itgα1 | Integrin α1 | 0.58 | 0.00033 |
| Thbs2 | Thrombospondin 2 | 0.57 | 0.00040 |
| Serpine1 | Serpin peptidase E1 | 0.57 | 0.00040 |
| Il1α | Interleukin 1α | 0.57 | 0.00045 |
| Il1rn | Interleukin 1 receptor antagonist | 0.57 | 0.00048 |
| Tgfβ2 | Transforming growth factor β2 | 0.56 | 0.00052 |
|  |  |  |  |

^1^The analysis is a correlation analysis between plasma TLR2 agonist and all measured features in the blockade and remission arms of the study (Figs 4 & 11) using Pattern Hunter in the MetaboAnalyst 3.0 statistical package.
