# Supplementary material for: Docosahexaenoic acid blocks progression of western diet-induced nonalcoholic steatohepatitis in obese Ldlr-/- mice
Source: PLoS One. 2017 Apr 19;12(4):e0173376. doi: 10.1371/journal.pone.0173376 (PMC5396882; doi:10.1371/journal.pone.0173376)
Supplement: S5 Table — (DOCX) [file pone.0173376.s005.docx]

**S5 Table:**

**Top 20 Features correlating with hepatic osteopontin (Opn) Expression: Blockage Arm^1^**

| **Feature** |  | **Correlation *r-value*** | ***p-value*** |
| --- | --- | --- | --- |
| **Mmp2** | Matrix metalloprotease 2 | 0.97 | 2.5 x 10^-13^ |
| **Plat** | Plasminogen activator-tissue | 0.97 | 3.2 x 10^-13^ |
| **Thbs2** | Thrombospondin 2 | 0.97 | 8.2 x 10^-13^ |
| **Itgβ5** | Integrin β5 | 0.96 | 4.5 x 10^-12^ |
| **Tgfβ2** | Transforming growth factor β2 | 0.96 | 8.3 x 10^-12^ |
| **Col3A1** | Collagen 3A1 | 0.96 | 1.2 x 10^-11^ |
| **Tgfβi1** | TGFβ induced homeobox 1 protein | 0.94 | 1.5 x 10^-10^ |
| **Timp2** | Tissue inhibitor metalloprotease 2 | 0.94 | 3.4 x 10^-10^ |
| **Ltbp1** | Latent transforming growth factor binding protein 1 | 0.94 | 4.5 x 10^-10^ |
| **Myc** | Myelocytomatosis oncogene | 0.93 | 1.2 x 10^-9^ |
| **Serpinh1** | Serpin peptidase inhibitor H1 | 0.92 | 2.7 x 10^-9^ |
| **Col1A2** | Collagen 1A2 | 0.92 | 5.5 x 10^-9^ |
| **Lox** | Lysyl oxidase | 0.91 | 7.3 x 10^-9^ |
| **Timp3** | Tissue inhibitor metalloprotease 2 | 0.91 | 8.3 x 10^-9^ |
| **Timp1** | Tissue inhibitor metalloprotease 2 | 0.89 | 9.2 x 10^-8^ |
| **Tgfβ3** | Transforming growth factor β3 | 0.89 | 9.3 x 10^-8^ |
| **Itgα3** | Integrin α3 | 0.89 | 9.7 x 10^-8^ |
| **Thbs1** | Thrombospondin 1 | 0.89 | 9.8 x 10^-8^ |
| **PdgfA** | Platelet derived growth factor-A | 0.88 | 9.9 x 10^-8^ |
| **Bcl2** | B-cell lymphoma 2 | 0.88 | 1.2 x 10^-7^ |

^1^The analysis is a correlation analysis between hepatic osteopontin expression with all measured features in the blockade arm of the study (Fig 4) using Pattern Hunter in the MetaboAnalyst 3.0 statistical package.
