# Supplementary material for: Docosahexaenoic acid blocks progression of western diet-induced nonalcoholic steatohepatitis in obese Ldlr-/- mice
Source: PLoS One. 2017 Apr 19;12(4):e0173376. doi: 10.1371/journal.pone.0173376 (PMC5396882; doi:10.1371/journal.pone.0173376)
Supplement: S6 Table — (DOCX) [file pone.0173376.s006.docx]

**S6 Table**

**Top 20 features correlating with hepatic osteopontin (Opn) expression: Remission Arm^1^**

|  |  |  |  |
| --- | --- | --- | --- |
| **Features** |  | **Correlation *r-value*** | ***p-value*** |
| **Ltbp1** | Latent transforming growth factor binding protein 1 | 0.97 | 0 |
| **Thbs2** | Thrombospondin 2 | 0.97 | 0 |
| **Col3a1** | Collagen 3A1 | 0.98 | 0 |
| **Mmp2** | Matrix metalloprotease 2 | 0.98 | 0 |
| **Plat** | Plasminogen activator-tissue | 0.98 | 0 |
| **Ccr2** | MCP1/Chemokine receptor 2 | 0.96 | 4.4 x 10^-16^ |
| **Itgβ5** | Integrin β5 | 0.96 | 1.3 x 10^-15^ |
| **Tgfβ2** | Transforming growth factor β2 | 0.96 | 1.3 x 10^-15^ |
| **Timp2** | Tissue inhibitor metalloprotease 2 | 0.95 | 2.0 x 10^-14^ |
| **Col1A2** | Collagen 1A2 | 0.94 | 1.3 x 10^-13^ |
| **Serpinh1** | Serpin peptidase inhibitor H1 | 0.93 | 3.3 x 10^-13^ |
| **Myc** | Myelocytomatosis oncogene | 0.93 | 1.0 x 10^-12^ |
| **Lox** | Lysyl oxidase | 0.92 | 2.4 x 10^-12^ |
| **LW** | Liver weight | 0.92 | 2.7 x 10^-12^ |
| **Bcl2** | B-cell lymphoma 2 | 0.92 | 3.2 x 10^-12^ |
| **Timp1** | Tissue inhibitor metalloprotease 1 | 0.92 | 6.7 x 10^-12^ |
| **Mmp1a** | Matrix metalloprotease 1a | 0.91 | 2.1 x 10^-11^ |
| **Tgfβ3** | Transforming growth factor β3 | 0.91 | 2.5 x 10^-11^ |
| **Thbs1** | Thrombospondin 1 | 0.90 | 5.8 x 10^-11^ |
| **LW%BW** | Liver weight % body weight | 0.89 | 1.5 x 10^-10^ |
|  |  |  |  |

^1^The analysis is a correlation analysis between hepatic osteopontin expression with all measured features in the remission arm of the study (Fig 11) using Pattern Hunter in the MetaboAnalyst 3.0 statistical package.
