# Supplementary material for: Docosahexaenoic acid blocks progression of western diet-induced nonalcoholic steatohepatitis in obese Ldlr-/- mice
Source: PLoS One. 2017 Apr 19;12(4):e0173376. doi: 10.1371/journal.pone.0173376 (PMC5396882; doi:10.1371/journal.pone.0173376)
Supplement: S7 Table — (DOCX) [file pone.0173376.s007.docx]

**S7 Table**

**Top 20 Features correlating with hepatic collagen 1A1 expression: Blockage Arm^1^**

| **Feature** |  | **Correlation *r-value*** | ***p-value*** |
| --- | --- | --- | --- |
| **pT Chol** | Plasma total cholesterol | 0.85 | 0 |
| **Tgfβ2** | Transforming growth factor β2 | 0.86 | 0 |
| **Gdf15** | Growth differentiation factor 2 | 0.84 | 0 |
| **Ccr2** | MCP1/Chemokine receptor 2 | 0.86 | 0 |
| **Cxcr4** | C-X-C chemokine receptor 4 | 0.84 | 0 |
| **Il12b** | Interleukin 12b | 0.85 | 0 |
| **Tnfsf12** | TNF superfamily 12 | 0.84 | 0 |
| **pTAG** | Plasma triglyceride | 0.90 | 2.4 x 10^-8^ |
| **ALT** | Alanine aminotransferase | 0.87 | 1.5 x 10^-8^ |
| **Tnfrsf11b** | TNF superfamily 11b | 0.87 | 1.5 x 10^-8^ |
| **20:1,ω9** | Gondolic acid | 0.87 | 1.5 x 10^-8^ |
| **AST** | Aspartate aminotransferase | 0.87 | 3.3 x 10^-8^ |
| **Il1α** | Interleukin 1α | 0.87 | 3.7 x 10^-8^ |
| **Tgfβr2** | Transforming growth factor β receptor 2 | 0.83 | 4.2 x 10^-8^ |
| **Lox** | Lysyl oxidase | 0.83 | 4.2 x 10^-8^ |
| **LW** | Liver weight | 0.87 | 5.9 x 10^-8^ |
| **Tnfsf13** | TNF superfamily 13 | 0.87 | 5.9 x 10^-8^ |
| **Col1A2** | Collagen 1A2 | 0.83 | 6.4 x 10^-8^ |
| **Mmp1a** | Matrix metalloprotease 1a | 0.88 | 1.0 x 10^-8^ |
| **TgFβi1** | TGFβ induced homeobox 1 protein | 0.83 | 1.1 x 10^-8^ |

^1^Correlation analysis between hepatic collagen 1A1 expression and all measured features in the blockade arm (Fig 4) using Pattern Hunter in the MetaboAnalyst 3.0 statistical package.
