# Supplementary material for: Docosahexaenoic acid blocks progression of western diet-induced nonalcoholic steatohepatitis in obese Ldlr-/- mice
Source: PLoS One. 2017 Apr 19;12(4):e0173376. doi: 10.1371/journal.pone.0173376 (PMC5396882; doi:10.1371/journal.pone.0173376)
Supplement: S8 Table — (DOCX) [file pone.0173376.s008.docx]

**S8 Table**

**Top 20 Features correlating with hepatic collagen 1A1 expression: Remission Arm^1^**

| **Features** |  | **Correlation *r-value*** | ***p-value*** |
| --- | --- | --- | --- |
| **Ccr2** | MCP1/Chemokine receptor 2 | 0.93 | 1.0 x 10^-12^ |
| **Thbs2** | Thrombospondin 2 | 0.92 | 5.0 x 10^-12^ |
| **Lox** | Lysyl oxidase | 0.92 | 6.4 x 10^-12^ |
| **Tnfsf13b** | TNF superfamily 13b | 0.91 | 2.0 x 10^-11^ |
| **Plat** | Plasminogen activator-tissue | 0.90 | 5.4 x 10^-11^ |
| **Mmp2** | Matrix metalloprotease 2 | 0.90 | 7.2 x 10^-11^ |
| **LW** | Liver weight | 0.90 | 1.1 x 10^-10^ |
| **Col3a1** | Collagen 3A1 | 0.90 | 1.2 x 10^-10^ |
| **Ltbp1** | Latent transforming growth factor binding protein 1 | 0.89 | 2.3 x 10^-10^ |
| **LW%BW** | Liver weight % Body weight | 0.89 | 3.0 x 10^-10^ |
| **Opn** | Osteopontin | 0.88 | 6.1 x 10^-10^ |
| **Serpinh1** | Serpin peptidase inhibitor H1 | 0.88 | 7.0 x 10^-10^ |
| **Tgfβ2** | Transforming growth factor β2 | 0.88 | 8.0 x 10^-10^ |
| **Myc** | Myelocytomatosis oncogene | 0.88 | 1.1 x 10^-09^ |
| **Thbs1** | Thrombospondin 1 | 0.87 | 1.4 x 10^-09^ |
| **Itgβ5** | Integrin β5 | 0.87 | 1.4 x 10^-09^ |
| **Tgfβ3** | Transforming growth factor β3 | 0.87 | 2.6 x 10^-09^ |
| **Timp2** | Tissue inhibitor metalloprotease 2 | 0.86 | 4.2 x 10^-09^ |
| **Mmp1a** | Matrix metalloprotease 1a | 0.86 | 5.4 x 10^-09^ |
| **Bcl2** | B-cell lymphoma 2 | 0.86 | 6.1 x 10^-09^ |

^1^Correlation analysis between hepatic collagen 1A1 expression and all measured features in the remission arm (Fig 11) using Pattern Hunter in the MetaboAnalyst 3.0 statistical package.
