# Supplementary figures and images for: Docosahexaenoic acid blocks progression of western diet-induced nonalcoholic steatohepatitis in obese Ldlr-/- mice
Source: PLoS One. 2017 Apr 19;12(4):e0173376. doi: 10.1371/journal.pone.0173376 (PMC5396882; doi:10.1371/journal.pone.0173376)

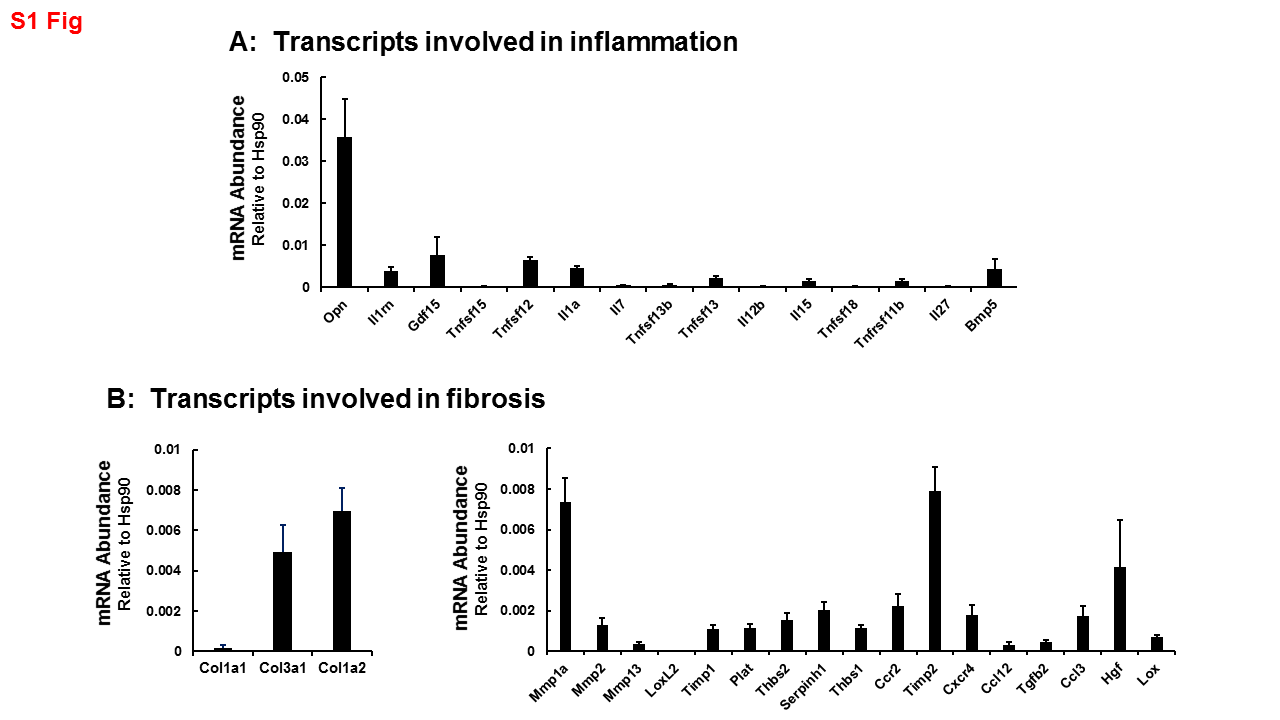

Supplement: S1 Fig — Relative abundance of hepatic transcripts linked to inflammation [A] and fibrosis [B]: Hepatic mRNA abundance was quantified using qRTPCR arrays as described in Materials and Methods. The reference gene was Hsp90. Results are represented as mRNA Abundance, Relative to Hsp90 (reference gene) for the RD group only. N = 5; mean ± SD. (TIF) [file pone.0173376.s009.tif]

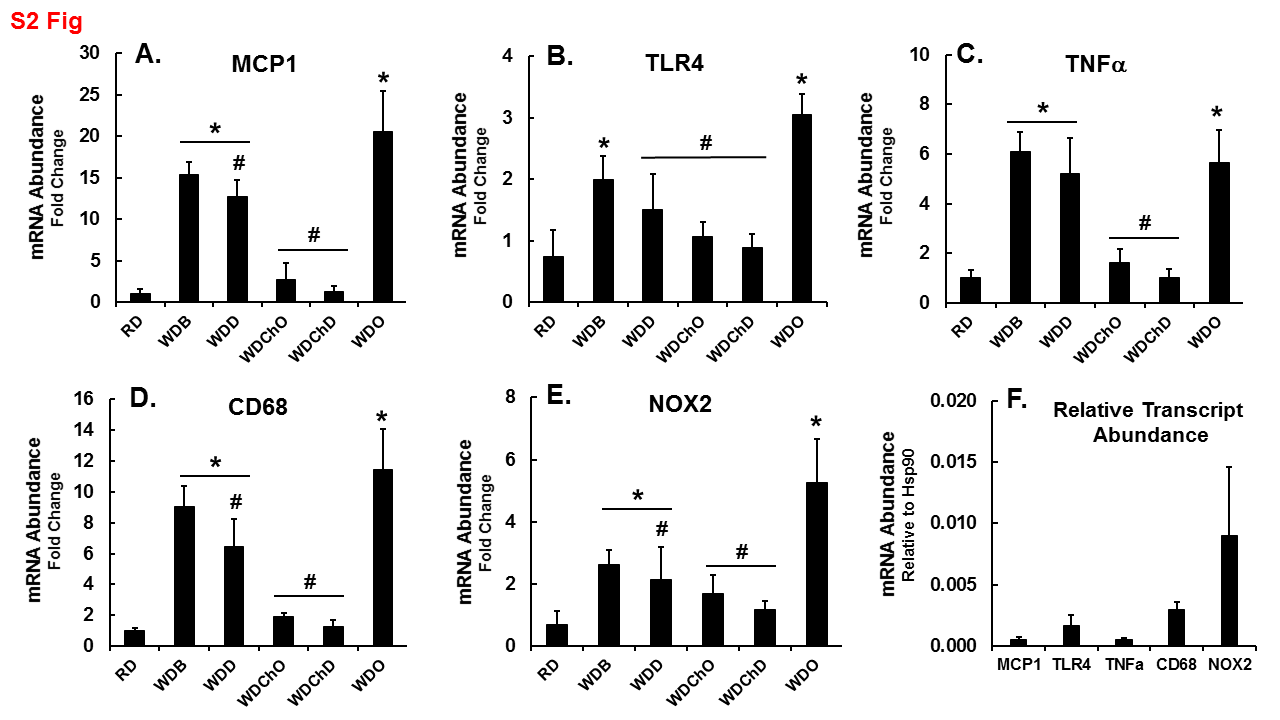

Supplement: S2 Fig — A-E: Hepatic mRNA abundance was quantified as described in Materials and Methods using in-house PCR primers. Results are represented as mRNA Abundance-Fold Change; N = 4–7; mean ± SD; *, p<0.05 versus the RD group; #, p<0.05 versus the WDO group; one-way ANOVA. F: Hepatic mRNA abundance was quantified using qRTPCR arrays as described in Materials and Methods. The reference gene was cyclophilin. Results are represented as mRNA Abundance, Relative to Cyclophilin (reference gene) for the RD group only. N = 5; mean ± SD. (TIF) [file pone.0173376.s010.tif]
